# Supplementary material for: Poor psychological health and 8-year mortality: a population-based prospective cohort study stratified by gender in Scania, Sweden
Source: BMJ Open. 2022 Nov 22;12(11):e056367. doi: 10.1136/bmjopen-2021-056367 (PMC9684964; doi:10.1136/bmjopen-2021-056367)
Supplement: Supplementary data [file bmjopen-2021-056367supp001.pdf]

Supplementary Table 1. Descriptive characteristics (%) of age, SES, sedentary leisure time, daily smoking, alcohol, BMI, and chronic distress by psychological distress (GHQ-12 ≥4).

The 2008 Public Health Survey of Scania, Sweden. Total population n=25503. Weighted prevalence.

	**Women**			**Men**		
	n = 13984			n = 11519		
	**Psychological distress**			**Psychological distress**		
	**No**	**Yes**	p-value	**No**	**Yes**	p-value
	n = 11950	n = 2034		n = 10283	n = 1236	
	83.8 %	**16.2 %**		87.9 %	**12.1 %**	
**Age**, yrs: mean ± SD [a]	46.6 ± 16.0	40.6 ± 16.6		47.2 ± 17.7	44.0± 17.5	
	(46.2-47.0)	(39.7-41.4)	<0.001	(46.8-47.6)	(42.9-45.0)	<0.001
**Body Mass Index (BMI)**:						
mean ± SD [a]	24.9 ± 4.6	24.9 ± 5.3		26.1 ± 4.1	26.4 ± 5.1	
	(24.8-25.1)	(24.7-25.2)	0.931	(26.0-26.2)	(26.0-26.7)	0.102
**Socioeconomic status (SES)** [b]						
Higher non-manual	7.9	6.8		9.8	9.4	
	(7.3-8.4)	(5.5-8.1)		(9.1-10.5)	(7.5-11.3)	
Medium non-manual	15.7	13.8		12.3	8.5	
	(15.0-16.5)	(12.0-15.6)		(11.5-13.1)	(6.7-10.3)	
Lower non-manual	10.6	9.6		5.3	5.1	
	(9.9-11.3)	(8.1-11.2)		(4.8-5.9)	(3.6-6.6)	
Skilled manual	9.5	7.6		12.0	10.1	
	(8.6-10.1)	(6.1-9.0)		(11.2-12.8)	(7.9-12.3)	
Unskilled manual	12.6	10.5		13.4	9.6	
	(11.8-13.3)	(8.9-12.1)		(12.5-14.2)	(7.5-11.6)	
Self-employed/farmer	4.1	3.1		8.3	6.4	
	(3.7-4.6)	(2.1-4.1)		(7.6-9.0)	(4.8-8.0)	
Early retired	3.9	7.3		2.6	10.1	
	(3.5-4.4)	(5.8-8.7)		(2.2-3.0)	(7.7-12.4)	
Unemployed	3.3	9.3		3.0	10.1	
	(2.8-3.7)	(7.6-11.0)		(2.5-3.5)	(7.9-12.4)	
Student	8.8	13.2		6.5	9.9	
	(8.1-9.5)	(11.1-15.3)		(5.9-7.2)	(7.5-12.3)	
Old age pensioner	18.6	9.9		19.9	10.1	
	(17.8-19.4)	(8.4-11.3)		(19.0-20.8)	(8.4-11.9)	
Unclassified	4.2	4.5		6.4	6.6	
	(3.7-4.7)	(3.3-5.7)		(5.7-7.1)	(4.6-8.5)	
Long-term sickleave	0.8	4.4		0.5	4.2	
	(0.6-1.0)	(3.3-5.6)	<0.001	(0.3-0.6)	(2.9-5.5)	<0.001
**Sedentary leisure time** [b]	11.2	21.4		13.7	28.9	
	(10.4-11.9)	(19.1-23.7)	<0.001	(12.8-14.6)	(25.6-32.1)	<0.001
**Daily smoking** [b]	14.7	19.6		12.7	22.1	
	(13.9–15.5)	(17.5-21.8)	<0.001	(11.8-13.5)	(19.1-25.1)	<0.001
**Alcohol drinking past year**[b]						
Never	14.2	18.6		8.6	14.8	
	(13.3-15.0)	(16.5-20.8)		(7.9-9.4)	(12.1-17.5)	
Once a month or more	27.1	28.6		18.2	23.4	
seldom	(26.1-28.1)	(26.1-31.0)		(17.3-19.2)	(20.4-26.5)	
2-4 times a month	34.6	31.5		37.8	29.5	
	(33.5-35.6)	(28.9-34.0)		(36.7-39.0)	(26.3-32.6)	
2-3 times a week	19.7	15.3		25.8	22.5	
	(18.9-20.6)	(13.5-17.2)		(24.8-26.8)	(19.6-25.4)	
At least 4 times a week	4.4	6.0		9.4	9.9	
	(4.0-4.9)	(4.8-7.1)	<0.001	(8.8-10.1)	(8.0-11.7)	<0.001
**Chronic disease*** [b]	26.8	45.3		24.8	47.8	
	(25.9-27.8)	(42.7-48.0)	<0.001	(23.8-25.8)	(44.4-51.2)	<0.001

GHQ-12 = 12 item version of the General Health Questionnaire, 0-12 points. Psychological distress was defined as GHQ-12≥4.
* Chronic disease = long-term disease, injury-related trouble, disability or other weakness.
[a] p-value: Independent samples T-test, 2-tailed
[b] p-value: Pearson Chi Square test, 2-sided
The values in parentheses are 95% confidence intervals for mean or percent based on bootstrap method with 2000 number of replicates.
