# Supplementary material for: Poor psychological health and 8-year mortality: a population-based prospective cohort study stratified by gender in Scania, Sweden
Source: BMJ Open. 2022 Nov 22;12(11):e056367. doi: 10.1136/bmjopen-2021-056367 (PMC9684964; doi:10.1136/bmjopen-2021-056367)
Supplement: Supplementary data [file bmjopen-2021-056367supp002.pdf]

Suppl Table 2. ORs from logistic regression models for all-cause mortality and cause-specific mortality, showing association with psychological distress (GHQ ≥4).
The 2008 Scania public health survey with 8.3 years follow-up.
Men and women combined; n = 25503.

	Model 0		Model 1		Model 2		
**Cause of death**	**OR**	**(95%CI)**	**OR**	**(95%CI)**	**OR**	**(95%CI)**	**Number of deaths**
**All causes**	**3.0*** **	(2.4-3.8)	**2.2*** **	(1.8-2.8)	**1.8*** **	(1.5-2.3)	1389
**Cause-specific:**							
**Cardiovascular**	**3.1*** **	(2.2-4.4)	**2.2*** **	(1.5-3.3)	**1.9*** **	(1.3-2.8)	425
**Cancer**	**1.8*** **	(1.3-2.4)	**1.5* **	(1.1-2.1)	1.3	(0.9-1.8)	539
**Other causes**	**3.1*** **	(2.2-4.2)	**2.1*** **	(1.5-2.9)	**1.7** **	(1.2-2.4)	425

Model 0 adjusted for age and gender.
Model 1 furthermore adjusted for socioeconomic status, physical activity, smoking, and alcohol.
Model 2 furthermore adjusted for chronic disease.
Significance levels: * p<0.05, ** p<0.01, *** p<0.001
Weighted Odds Ratios. Bootstrap method (2000 replicates) for variation estimation.
