# Supplementary material for: Poor psychological health and 8-year mortality: a population-based prospective cohort study stratified by gender in Scania, Sweden
Source: BMJ Open. 2022 Nov 22;12(11):e056367. doi: 10.1136/bmjopen-2021-056367 (PMC9684964; doi:10.1136/bmjopen-2021-056367)
Supplement: Supplementary data [file bmjopen-2021-056367supp003.pdf]

Suppl Table 3. ORs from logistic regression models for all-cause mortality and cause-specific mortality, showing association with psychological distress (GHQ ≥4).

The 2008 Scania public health survey with 8.3 years follow-up.

Stratified by gender; n = 13984 women and 11519 men.

	Model 0		Model 1		Model 2		
**Cause of death**	**OR**	**(95%CI)**	**OR**	**(95%CI)**	**OR**	**(95%CI)**	**Number of deaths**
**All causes**							
Women	**2.5*** **	(1.9-3.4)	**1.7****	(1.2-2.3)	**1.4***	(1.1-2.0)	574
Men	**3.5*****	(2.6-4.7)	**2.7*****	(2.0-3.7)	**2.2*****	(1.6-3.0)	815
**Cause-specific:**							
**Cardiovascular**							
Women	**2.7*****	(1.6-4.6)	1.7	(1.0-2.9)	1.4	(0.8-2.5)	140
Men	**3.3*****	(2.1-5.2)	**2.5*****	(1.5-4.2)	**2.1****	(1.3-3.5)	285
**Cancer**							
Women	1.5	(0.9-2.3)	1.1	(0.7-1.9)	1.0	(0.6-1.7)	258
Men	**2.1*****	(1.3-3.3)	**1.9****	(1.2-2.9)	**1.6***	(1.0-2.6)	281
**Other causes**							
Women	**3.0*****	(1.9-4.8)	**1.9***	(1.2-3-0)	1.6	(1.0-2.5)	176
Men	**3.0*****	(1.9-4.7)	**2.2*****	(1.4-3.4)	**1.8***	(1.1-3.0)	249

Model 0 adjusted for age.

Model 1 furthermore adjusted for socioeconomic status, physical activity, smoking, and alcohol.

Model 2 furthermore adjusted for chronic disease.

Significance levels: * p<0.05, ** p<0.01, *** p<0.001

Weighted Odds Ratios. Bootstrap method (2000 replicates) for variation estimation.
