# Supplementary material for: Poor psychological health and 8-year mortality: a population-based prospective cohort study stratified by gender in Scania, Sweden
Source: BMJ Open. 2022 Nov 22;12(11):e056367. doi: 10.1136/bmjopen-2021-056367 (PMC9684964; doi:10.1136/bmjopen-2021-056367)
Supplement: Supplementary data [file bmjopen-2021-056367supp004.pdf]

Suppl Table 4. Associations between different levels of psychological distress (GHQ-12) and all-cause mortality.
The 2008 Scania public health survey with 8.3 years follow-up.
Total population (n=25503) and stratified by gender (n=13984 women and 11519 men).

GHQ-score	Model 0 OR	(95%CI)	Model 1 OR	(95%CI)	Model 2 OR	(95%CI)	Number of deaths
**Total population**							
0=REF	1.0		1.0		1.0		1389
1-3	**1.8*** **	(1.5-2.2)	**1.5*** **	(1.2-1.9)	**1.4** **	(1.1-1.7)	
4-6	**2.8*** **	(2.1-3.7)	**2.1*** **	(1.5-2.8)	**1.7** **	(1.3-2.4)	
7-12	**4.0*** **	(3.0-5.3)	**2.8*** **	(2.1-3.7)	**2.2*** **	(1.6-3.0)	
**Women**							
0=REF	1.0		1.0		1.0		574
1-3	**1.7*** **	(1.3-2.3)	**1.4* **	(1.0-1.9)	1.3	(0.9-1.7)	
4-6	**2.9*** **	(1.9-4.3)	**2.0** **	(1.3-3.1)	**1.7* **	(1.1-2.6)	
7-12	**2.7*** **	(1.9-4.0)	**1.7** **	(1.2-2.5)	1.4	(1.0-2.1)	
**Men**							
0=REF	1.0		1.0		1.0		815
1-3	**1.9*** **	(1.4-2.5)	**1.6*** **	(1.2-2.2)	**1.4* **	(1.1-1.9)	
4-6	**2.7*** **	(1.8-4.0)	**2.1*** **	(1.4-3.3)	**1.8** **	(1.2-2.7)	
7-12	**5.1*** **	(3.5-7.6)	**3.8*** **	(2.5-5.7)	**3.0*** **	(1.9-4.6)	

Model 0 adjusted for age (and gender in analysis of total population).
Model 1 furthermore adjusted for socioeconomic status, physical activity, smoking, and alcohol.
Model 2 furthermore adjusted for chronic disease.
Significance levels: * $p<0.05$, ** $p<0.01$, *** $p<0.001$
Weighted Odds Ratios. Bootstrap method (2000 replicates) for variation estimation.
