# Supplementary material for: Poor psychological health and 8-year mortality: a population-based prospective cohort study stratified by gender in Scania, Sweden
Source: BMJ Open. 2022 Nov 22;12(11):e056367. doi: 10.1136/bmjopen-2021-056367 (PMC9684964; doi:10.1136/bmjopen-2021-056367)
Supplement: Author's manuscript [file bmjopen-2021-056367.draft_revisions.pdf]

BMJ Open

BMJ Open is committed to open peer review. As part of this commitment we make the peer review history of every article we publish publicly available.

When an article is published we post the peer reviewers' comments and the authors' responses online. We also post the versions of the paper that were used during peer review. These are the versions that the peer review comments apply to.

The versions of the paper that follow are the versions that were submitted during the peer review process. They are not the versions of record or the final published versions. They should not be cited or distributed as the published version of this manuscript.

BMJ Open is an open access journal and the full, final, typeset and author-corrected version of record of the manuscript is available on our site with no access controls, subscription charges or pay-per-view fees (http://bmjopen.bmj.com).

If you have any questions on BMJ Open's open peer review process please email info.bmjopen@bmj.com

Poor psychological health and 5-year mortality: A population-based prospective cohort study stratified by gender in Scania, Sweden

Journal:	*BMJ Open*
Manuscript ID	bmjopen-2021-056367
Article Type:	Original research
Date Submitted by the Author:	16-Aug-2021

Complete List of Authors:	Fridh, Maria; Lund University Faculty of Medicine, Social Medicine and Health Policy, Department of Clinical Sciences in Malmö<br>Pirouzifard, Mirnabi; Lund University Faculty of Medicine, Social Medicine and Health Policy, Department of Clinical Sciences in Malmö; Region Skåne and Lund University, Centre for Primary Health Care Research<br>Rosvall, Maria; Lund University Faculty of Medicine, Social Medicine and Health Policy, Department of Clinical Sciences in Malmö; University of Gothenburg Sahlgrenska Academy, School of Public Health, Institute of Medicine<br>Lindstrom, Martin; Lund University Faculty of Medicine, Social Medicine and Health Policy, Department of Clinical Sciences in Malmö; Region Skåne and Lund University, Centre for Primary Health Care Research

Keywords:	PUBLIC HEALTH, EPIDEMIOLOGY, MENTAL HEALTH, SOCIAL MEDICINE

I, the Submitting Author has the right to grant and does grant on behalf of all authors of the Work (as defined in the below author licence), an exclusive licence and/or a non-exclusive licence for contributions from authors who are: i) UK Crown employees; ii) where BMJ has agreed a CC-BY licence shall apply, and/or iii) in accordance with the terms applicable for US Federal Government officers or employees acting as part of their official duties; on a worldwide, perpetual, irrevocable, royalty-free basis to BMJ Publishing Group Ltd ("BMJ") its licensees and where the relevant Journal is co-owned by BMJ to the co-owners of the Journal, to publish the Work in this journal and any other BMJ products and to exploit all rights, as set out in our licence.

The Submitting Author accepts and understands that any supply made under these terms is made by BMJ to the Submitting Author unless you are acting as an employee on behalf of your employer or a postgraduate student of an affiliated institution which is paying any applicable article publishing charge ("APC") for Open Access articles. Where the Submitting Author wishes to make the Work available on an Open Access basis (and intends to pay the relevant APC), the terms of reuse of such Open Access shall be governed by a Creative Commons licence – details of these licences and which Creative Commons licence will apply to this Work are set out in our licence referred to above.

Other than as permitted in any relevant BMJ Author's Self Archiving Policies, I confirm this Work has not been accepted for publication elsewhere, is not being considered for publication elsewhere and does not duplicate material already published. I confirm all authors consent to publication of this Work and authorise the granting of this licence.

For peer review only

Poor psychological health and 5-year mortality: A population-based prospective cohort study stratified by gender in Scania, Sweden

Maria Fridh[1]*, MD, PhD, **Mirnabi Pirouzifard [1,2], PhD**, Maria Rosvall[1,3,4], MD, PhD, and Martin Lindström[1,2], MD, PhD

[1] Social Medicine and Health Policy, Department of Clinical Sciences in Malmö, Lund University, CRC, Jan Waldenströmsgata 35, Skåne University Hospital Malmö, SE-205 02 Malmö, Sweden.

[2] Centre for Primary Health Care Research, Region Skåne and Lund University, S-205 02 Malmö, Sweden.

[3] **School of Public Health, Institute of Medicine, Sahlgrenska academy,** University of Gothenburg, Sweden.

[4] **Socialmedicinskt centrum, Regionhälsan, Västra Götalandsregionen,** Sweden.

E-mail:

Maria Fridh,* maria.fridh@med.lu.se

Mirnabi Pirouzifard, mirnabi.pirouzifard@med.lu.se

Maria Rosvall, maria.rosvall@gu.se

Martin Lindström, martin.lindstrom@med.lu.se

* Corresponding author

For peer review only

WORD COUNT: 4000

ABSTRACT

Objectives: We investigated gender differences in the association between mortality and general psychological distress (**measured** by GHQ-12), as an increased mortality risk has been shown in community studies, but gender differences are largely unknown. **Setting**: We used data from a cross-sectional population-based public health survey conducted in 2008 in the Swedish region of Skåne (Scania) **of people 18-80 years old** (response rate 54.1 %). The relationship between psychological distress and subsequent all-cause and cause-specific mortality was examined by Cox proportional hazard models for the total study population and stratified by gender, adjusting for age, socioeconomic status, life-style (physical activity, smoking, alcohol consumption) and chronic disease. **Participants**: Of 28198 respondents, **25503** were included **in analysis by restrictive criteria**. **Outcome measures**: Overall and cause-specific mortality by December 31st, 2013. **Results**: More women (**20.2** %) than men (**15.7** %) reported psychological distress at baseline (GHQ ≥3). During a mean follow-up of 5.2 years, **775** participants died: **230** **(29.7%)** from cardiovascular diseases, **324 (41.8%)** from cancer, and **221 (28.5%)** from other causes. The overall association between psychological distress and mortality risk held for all mortality end-points after multiple adjustments (e.g. all-cause mortality HR: **1.8 (95 % CI: 1.5 – 2.3) for men and women combined.** However, stratification revealed a clear gender difference as **the association between GHQ-12 and mortality was strong and robust through all adjustments for men but not for women**. **Conclusion: More** women than men **reported** psychological distress **while** mortality was higher among men (i.e. the morbidity-mortality gender paradox)**.** GHQ-12 could potentially be used as one of several predictors of mortality, **especially for men. In the future, screening tools for psychological distress should be validated for both men**

For peer review only - http://bmjopen.bmj.com/site/about/guidelines.xhtml

and women. Further research regarding the underlying mechanisms of the gender paradox is warranted.

KEY WORDS: Mortality, Psychological Distress, Prospective Study, Sweden, GHQ-12, Gender Paradox

Strengths and limitations of this study

- This was a large, prospective, randomly selected stratified population-based study with valid exposure and outcome measures (GHQ-12 and mortality).
- The participant rate was moderately high with no substantial selection bias.
- Relevant potential confounders (age, SES, physical activity, smoking, alcohol consumption) were included in the multiple statistical models.
- In order to investigate reverse causality, the final model adjusted for self-reported chronic disease, which may be a less valid indicator than objective measures of pre-existing disease at baseline.
- Although statistical models included several potentially relevant confounders, residual confounding cannot be excluded.

INTRODUCTION

Increasing psychological distress in the general population, especially among young people, is of great public health concern in Sweden.[1] **Unspecified psychological distress has been shown to have a high and persistent mortality effect in the general population independently of clinical depression and other severe mental illness.[2] The mechanisms behind this are probably both biological and behavioural with intricate interactions across pathways, contributing to e.g. cardiovascular disease, cancer progression and premature mortality.[3, 4]**

For peer review only - http://bmjopen.bmj.com/site/about/guidelines.xhtml

For peer review only

The General Health Questionnaire is an extensively used, reliable and validated measure of unspecific psychological distress in population studies,[5, 6] and in Sweden the 12-item General Health Questionnaire (GHQ-12) has been used for decades in national and regional surveys.[7] Earlier population-based research has demonstrated an association between **psychological** distress measured by the GHQ-12 and risk of all-cause mortality,[8-11] as well as cause-specific mortality (e.g. cardiovascular death,[8, 10-14] cancer,[10, 15, 16] **and other causes**.[11]) **The association with GHQ-12 is clear and robust for all-cause mortality and cardiovascular mortality, but mixed for cancer mortality (some studies show an association with higher levels of psychological distress for all-cancer mortality,[10] some for certain types of cancer only,[15, 16] and other studies no association at all.[11])** One confounder of the relationship between psychological distress and premature death might be the presence of pre-existing disease at baseline (contributing both to distress and subsequent higher mortality rates, i.e. ”reverse causality”).[10] Several studies on GHQ-12 and mortality attempted to evaluate the possibility of reverse causality by adjusting for different measures of baseline physical illness, and/or excluding all deaths the first years of follow-up. **Reverse causality was not found to explain the association between GHQ-12 and all-cause mortality or cardiovascular mortality,[8, 10, 11, 13, 14] but results were less clear for cancer mortality.[10, 15, 16]** A dose-response relationship between psychological distress and increased risk of mortality may support the existence of a causal relationship,[8] and several studies reported a dose-response pattern **for all-cause mortality and cardiovascular mortality**[8-11, 13, 14] (some very large studies showed an increased mortality risk even at lower levels of distress).[10, 14**] Results on dose-response for cancer mortality were less consistent.[10, 16]**

For peer review only - http://bmjopen.bmj.com/site/about/guidelines.xhtml

The fact that psychological distress is more often reported by women than men while mortality rates are higher among men is an example of the male-female health-survival paradox.[17-19] Women are in general more likely to acquire disabling conditions such as arthritis, autoimmune diseases and depression, while men are more likely to acquire lethal conditions at younger ages, such as lung cancer and myocardial infarction.[17-19] There are multiple explanations for the gender paradox, but it is thought that both sex (biological factors) and gender (social factors) play important and interacting roles.[18] The most prominent biological explanations are *genetic* (two X-chromosomes constitute a survival/ageing advantage), *hormonal* (e.g. the protective effect of estrogens on serum lipids delaying atherosclerosis in females), and *immunological* (testosterone may reduce the robustness of the male immunological system with a higher risk of life-threatening infections, while immunological effects of estrogens may increase the risk of autoimmunity and chronic inflammation).[19, 20] Social explanations focus on differences in *behavior and social roles*. Men are more likely than women to engage in health damaging risk-taking behaviours such as excessive alcohol consumption, smoking, drug use and unsafe driving. Gender roles may also contribute to differences in willingness and ability to adopt a sick role, seek help and access health care (men are more likely to present late with symptoms).[18-20] Despite decades of research, it is still not fully understood whether behavioural factors explain most of the gender gap or whether biological and social differences contribute more substantially to the gender gap in health and mortality.[19]

Gender differences in the association between psychological distress measured by GHQ-12 and mortality have rarely been investigated. In our literature search we found only one study that specifically aimed to examine gender-specific associations between GHQ-

For peer review only - http://bmjopen.bmj.com/site/about/guidelines.xhtml

12 and all-cause mortality.[9] In this study from Finland, 923 persons (414 men and 509 women) answered GHQ-12 at baseline and were followed during a mean observation time of 11 years with 44 death events (27 men, 17 women). Adjustments were made for gender, age, socioeconomic status, BMI, smoking and physical activity. The hazard ratio (HR) increased for every GHQ-12 point. The 10-year survival for distressed (GHQ ≥4) vs non-distressed (GHQ <4) men was significantly shorter (HR=3.38 (95%CI: 1.55-7.39) but there was no significant difference in survival between distressed and non-distressed women. The authors concluded that the increased all-cause mortality risk among the psychologically distressed was mainly due to excess mortality among distressed men. However, a weaker significant association among women may not have been detected due to lack of statistical power.

Using a large representative population from the Swedish region of Skåne (Scania) with baseline GHQ-12 (survey data) and 5-year prospective mortality data, the present study aimed to:

1) investigate the association between psychological distress and mortality (all-cause and cause-specific) including adjustment for self-reported chronic disease (as an indicator of potential reverse causality); and

2) explore a dose-response effect of psychological distress on all-cause mortality.

Furthermore, as an additional minor aim we wanted to compare two different GHQ-12 cut-off scores: GHQ ≥3 (conventionally used in Swedish public health surveys)[7] and GHQ ≥4 (widely used internationally).[6, 12, 15]

All analyses were performed on men and women combined and furthermore stratified by gender.

For peer review only - http://bmjopen.bmj.com/site/about/guidelines.xhtml

METHODS

Participants and study design

Data from the cross-sectional 2008 Scania public health survey was used. A total of 52142 persons aged 18–80 years (a random stratified sample selected from the official population registers of people living in Scania including 5.8% of the total population 18-80 years old) were invited to participate. The first information letter was sent out on August 27, 2008 with the option of answering the survey online. The paper questionnaires were sent by **regular** mail on September 5, as well as three reminders. A total of 28198 persons returned completed questionnaires (response rate 54.1 %**) Comparison between participants with register statistics of the total population in Scania showed that the age interval 18-34 years was somewhat under-represented and the age interval 65-80 years somewhat over-represented. Some under-representation of men and persons with low formal education was also observed, but the most serious under-representation concerned people not born in Europe.** To compensate for selection bias, the geographically stratified random sample was weighted by age, sex, country of birth, marital status, income, and education through **a weighting variable designed by Statistics Sweden (SCB)**.

We linked study participants to mortality data and followed them until December 31st, 2013. Of the 28198 respondents in the survey, 136 could not be traced, leaving a cohort of 28062 persons. **The study sample was restricted to those with no missing values in covariates used, i.e.; 25503 individuals, 11519 men (45%) and 13984 women (55%).**

Patient and Public Involvement statement (PPI)

The present study was based on **data from** a community population **survey** in the Swedish region of Scania. **The public was not involved in the design of this study. As the Swedish person numbers were not included in the data delivery, participants cannot be**

For peer review only - http://bmjopen.bmj.com/site/about/guidelines.xhtml

individually identified. Results of the present study will be disseminated to the public through publication in scientific journals.

Predictor and outcome variables

Psychological distress was assessed by the 12-item General Health Questionnaire (GHQ-12).[5, 6] The twelve items reflect different aspects of psychological health such as anxiety, depression, sleep disturbance, loss of confidence and the ability to perform daily activities and cope with everyday problems during **a time-frame of** ”the past few weeks”. **Cronbach coefficient alpha showed good internal consistency (men: 0.893; women: 0.896).** Interpretation of answers is based on a 4-point response scale scored using a bimodal method (symptom present: not at all = 0, same as usual = 0, more than usual = 1, much more than usual = 1) resulting in a GHQ-score of 0-12. **Mean GHQ-score was 1.49 for men and 1.61 for women ($p<0.001$).** We used the Swedish conventional score of ≥ 3 to define psychological distress.[7] To determine whether there was a dose-response relation, we categorized the results from the GHQ-12 into four groups (no distress = 0; sub-clinical distress = 1-2; moderate distress = 3-5; and high distress = 6-12).

Mortality was assessed from August 27, 2008 to December 31, 2013, which amounts to a **maximum period of 5.34 years. (Mean value men: 5.18 years; women: 5.22 years. Median value men and women both 5.28 years).** Cause of death was assessed according to ICD-10 and subdivided into three mortality categories: 1) cardiovascular causes of death (e.g. myocardial infarction and stroke, ICD 1109-1729), 2) death from cancer (C019-C979) and 3) death from other causes (A047-B999, D329-G931, J101-/869). **Information regarding mortality and cause of death was obtained from The National Cause of Death Register (Dödsorsaksregistret, Socialstyrelsen).**

For peer review only - http://bmjopen.bmj.com/site/about/guidelines.xhtml

Covariates

The following covariates were chosen as they are both known predictors of mortality and associated with psychological distress. *Age* was used as a **continuous** variable. *Socioeconomic status* (SES) was defined by 12 categories **of employment**: higher non-manual; medium non-manual; lower non-manual; skilled manual; unskilled manual; self-employed/farmer; early retired (before age 65 years, for health reasons or entitlement); unemployed; student, old-age pensioner (≥ 65 years of age); unclassified; and long-term sick-leave. **The participants' age and SES were registry data from Statistics Sweden (SCB) while all other covariates were self-reported data from the Scania public health survey**. Leisure-time *physical activity* was assessed from four alternatives, of which the **lowest level, sedentary** (walking, bicycling etc. less than 2 hours per week) **was dichotomized as "yes" and all other options as "no". Smoking was assessed by three categories: daily; intermittent/ non-daily; non-smoker, of which *daily smoking* was dichotomized as "yes" and all other options as "no".** Frequency of *alcohol consumption* past year was assessed by five categories. *Body Mass Index* (BMI) was based on self-reported data and did not differ between distressed and non-distressed men and women, **respectively**. As there was no significant age-gender adjusted association between BMI and mortality in our study sample, BMI was excluded from the multiadjusted analyses. *Chronic disease* was assessed by the question: "Do you have any long-term disease, injury-related trouble, disability or other weakness?" (yes/no).

Statistics

Chi-square and *t* tests were used to compare the sample characteristic differences between non-distressed/ **distressed, men and women respectively** (cut-off GHQ 2/3) (Table 1).

For peer review only - http://bmjopen.bmj.com/site/about/guidelines.xhtml

As the main aim of the study was to test gender differences, an initial test for effect modification by gender was performed. The interaction term between psychological distress and sex was significant (Hazard Ratio (HR) = 0.6; P = 0.002) indicating that the effect of psychological distress on mortality was different for men and women.

The proportional hazards assumption was considered fulfilled after inspection of the survival curves according to psychological distress (yes/no). Cox proportional hazards models were used to compute study specific hazard ratios with accompanying 95 % confidence intervals for the association between distress and all-cause mortality, as well as the association between distress and death caused by cardiovascular disease, cancer, or other causes, respectively. Model 0 adjusted for age and gender, Model 1 furthermore adjusted for SES, **low** physical activity, **daily** smoking, and alcohol **consumption**, and Model 2 furthermore adjusted for chronic disease. Table 2 shows Cox proportional hazards models for women and men combined. Table 3 shows Cox proportional hazards models stratified by gender (Model 0 age-adjusted only). Table 4 shows overall and gender-stratified associations between different levels of psychological distress and all-cause mortality (GHQ score: 0, 1-2, 3-5, 6-12). The **age-adjusted survival curves** of non-distressed (GHQ-12 <3) and distressed (GHQ-12 ≥ 3) participants, divided by gender, are shown in Figure 1. The statistical analyses were performed using **SAS 9.4, and all analyses were performed on weighted data. To ensure appropriate variance estimation on weighted data, we used bootstrap methods with 2000 numbers of replicates to obtain confidence intervals and p-values. The survey means procedure was used to obtain weighted descriptive statistics for continuous variables, and the survey frequency procedure was used for weighted one-way and multi-way crosstabulations. The survey phreg was used for weighted proportional hazard regression analysis.[21]**

For peer review only - http://bmjopen.bmj.com/site/about/guidelines.xhtml

In order to compare two different cut-off scores, identical statistical analyses using cut-off GHQ 3/4 were furthermore performed (see Supplementary Tables 1-4).

RESULTS

At baseline, more women than men were psychologically distressed, **20.2** % compared to **15.7** % (Table 1). Distressed study members were in average younger, more likely to not belong to the workforce, more likely to be sedentary, to smoke and to have a chronic disease. **As can be seen by non-overlapping confidence intervals, distressed women were significantly younger, leaner, less sedentary, and drank alcohol less often than distressed men.**

For peer review only - http://bmjopen.bmj.com/site/about/guidelines.xhtml

Table 1. Descriptive characteristics (%) of age, SES, sedentary leisure time, daily smoking, alcohol, BMI, and chronic distress by psychological distress (GHQ-12 ≥3).
The 2008 Public Health Survey of Scania, Sweden. Total population n=25503. Weighted prevalence.

	Women n = 13984 Psychological distress			Men n = 11519 Psychological distress		
	**No** n = 11452 79.8 %	**Yes** n = 2532 **20.2 %**	p-value	**No** n = 9925 84.3 %	**Yes** n = 1594 **15.7 %**	p-value
**Age**, yrs: mean ± SD [a]	46.9 ± 15.9 (46.5-47.3)	40.6 ± 16.6 (39.8-41.4)	<0.001	47.5 ± 17.6 (47.0-47.9)	43.4± 17.5 (42.4-44.3)	<0.001
**Body Mass Index (BMI)**: mean ± SD [a]	24.9 ± 4.6 (24.8-25.1)	24.9 ± 5.2 (24.7-25.2)	0.947	26.1 ± 4.1 (26.0-26.2)	26.3 ± 5.0 (26.0-26.6)	0.165
**Socioeconomic status (SES)** [b]						
Higher non-manual	7.8 (7.3-8.4)	7.2 (6.0-8.3)		9.9 (9.2-10.6)	9.0 (7.3-10.7)	
Medium non-manual	15.9 (15.1-16.7)	13.6 (11.9-15.2)		12.3 (11.5-13.2)	9.3 (7.8-10.9)	
Lower non-manual	10.6 (10.0-11.3)	9.6 (8.2-11.0)		5.3 (4.7-5.9)	5.3 (3.9-6.6)	
Skilled manual	9.6 (8.9-10.3)	7.7 (6.4-9.0)		11.8 (11.0-12.6)	11.3 (9.3-13.3)	
Unskilled manual	12.4 (11.6-13.2)	11.5 (10.0-12.9)		13.5 (12.6-14.4)	9.8 (8.1-11.2)	
Self-employed/farmer	4.1 (3.7-4.6)	3.2 (2.3-4.0)		8.4 (7.8-9.1)	6.1 (4.8-7.4)	
Early retired	3.9 (3.5-4.3)	6.7 (5.5-7.9)		2.6 (2.2-3.0)	8.7 (6.9-10.6)	
Unemployed	3.1 (2.6-3.6)	8.8 (7.3-10.2)		2.9 (2.4-3.3)	9.3 (7.4-11.2)	
Student	8.5 (7.8-9.2)	13.5 (11.7-15.4)		6.4 (5.7-7.1)	9.8 (7.8-11.7)	
Old age pensioner	19.1 (18.2-19.9)	9.7 (8.3-11.0)		20.3 (19.5-21.2)	10.0 (8.4-11.5)	
Unclassified	4.2 (3.6-4.7)	4.6 (3.5-5.7)		6.1 (5.4-6.8)	7.9 (6.0-9.7)	
Long-term sickleave	0.7 (0.5-0.9)	4.0 (3.0-5.0)	<0.001	0.4 (0.3-0.6)	3.5 (2.5-4.6)	<0.001
**Sedentary leisure time** [b]	11.0 (10.3-11.8)	19.8 (17.8-21.8)	<0.001	13.4 (12.6-14.3)	27.1 (24.3-29.9)	<0.001
**Daily smoking** [b]	14.6 (13.8–15.4)	19.2 (17.2-21.2)	<0.001	12.5 (11.6-13.3)	21.0 (18.3-23.7)	<0.001
**Alcohol drinking past year**[b]						
Never	14.0 (13.1-14.8)	18.6 (16.7-20.6)		8.6 (7.9-9.3)	13.6 (11.3-15.9)	
Once a month or more seldom	27.2 (26.2-28.2)	27.8 (25.6-30.0)		18.2 (17.2-19.1)	22.7 (20.0-25.3)	
2-4 times a month	34.4 (33.3-35.5)	32.8 (30.6-35.1)		37.7 (36.5-38.8)	32.3 (29.4-35.2)	
2-3 times a week	20.0 (19.1-20.9)	15.2 (13.5-16.9)		26.1 (25.0-27.1)	22.0 (19.4-24.5)	
At least 4 times a week	4.5 (4.0-4.9)	5.6 (4.6-6.6)	<0.001	9.5 (8.9-10.2)	9.4 (7.8-11.1)	<0.001
**Chronic disease*** [b]	26.5 (25.5-27.4)	43.1 (40.7-45.5)	<0.001	24.3 (23.2-25.3)	45.5 (42.4-48.5)	<0.001

GHQ-12 = Twelve item version of the General Health Questionnaire, 0-12 points. Psychological distress was defined as GHQ-12 ≥3.
* Chronic disease = long-term disease, injury-related trouble, disability or other weakness.
[a] p-value: Independent samples T-test, 2-tailed
[b] p-value: Pearson Chi Square test, 2-sided
The values in parentheses are 95% confidence intervals for mean or percent based on bootstrap method with 2000 number of replicates.

For peer review only - http://bmjopen.bmj.com/site/about/guidelines.xhtml

The mortality rates (# of deaths per 100 person-years) were calculated for men and women (data not shown). The overall mortality rate was 0.78 (95% CI: 0.71-0.85) for men and 0.43 (0.38-0.48) for women, with a male to female Incidence Rate Ratio (IRR) of 1.83 (95% CI: 1.58-2.11; p-value <0.001). The male to female IRR for cause-specific mortality were as follows: cardiovascular causes 2.98 (2.24-3.96; <0.001), cancer 1.32 (1.06-1.64; 0.013), and "other causes" 1.85 (1.41 – 2.42; <0.001). The male and female 95% confidence intervals were non-overlapping for all mortality outcomes except for cancer mortality where a minimal overlap was seen (95% CI: men 0.24-0.33, women 0.18-0.25).

Table 2 shows associations between psychological distress and mortality (all-cause and cause-specific) in multi-adjusted cox regression analyses for men and women combined. Age and gender-adjusted associations remained significant through Model 1 (SES, physical activity, smoking, alcohol) and Model 2 (chronic disease) for all mortality end-points. For all-cause mortality, the initial age-and gender adjusted hazard ratio 2.9 (95 % CI: 2.4 – 3.6) decreased to 1.8 (1.5 – 2.3) in the final model.

For peer review only - http://bmjopen.bmj.com/site/about/guidelines.xhtml

Table 2. HRs from Cox regression models for all-cause mortality and cause-specific mortality, showing association with psychological distress (GHQ ≥3).
The 2008 Scania public health survey with 5.3 years follow-up. Men and women combined, n=25503.

	Model 0		Model 1		Model 2		
**Cause of death**	**HR**	**(95%CI)**	**HR**	**(95%CI)**	**HR**	**(95%CI)**	**Number of deaths**
**All causes**	**2.9*** **	(2.4-3.6)	**2.1*** **	(1.7-2.7)	**1.8*** **	(1.5-2.3)	775
**Cause-specific:**							
**Cardiovascular**	**3.2*** **	(2.2-4.7)	**2.4*** **	(1.6-3.6)	**2.0*** **	(1.3-3.0)	230
**Cancer**	**2.6*** **	(1.9-3.6)	**2.0*** **	(1.4-2.8)	**1.7** **	(1.2-2.5)	324
**Other causes**	**3.1*** **	(2.1-4.6)	**2.1*** **	(1.4-3.1)	**1.7* **	(1.1-2.7)	221

Model 0 adjusted for age and gender.
Model 1 furthermore adjusted for socioeconomic status, physical activity, smoking, and alcohol.
Model 2 furthermore adjusted for chronic disease.
Significance levels: * $p<0.05$, ** $p<0.01$, *** $p<0.001$
Weighted Hazard Ratios. Bootstrap method (2000 replicates) for variation estimation.

Further analysis revealed a clear gender difference (Table 3), as the association between psychological distress and mortality remained robust for all mortality-outcomes through the modelling among men, but lost significance among women in Model 1. The age-adjusted hazard ratio for all-cause mortality decreased from 3.6 (2.7 – 4.7) to 2.4 (1.8 – 3.2) in men, and from 2.1 (1.5 – 2.8) to 1.2 (0.9 – 1.6) in women after full adjustments.

For peer review only - http://bmjopen.bmj.com/site/about/guidelines.xhtml

Table 3. HRs from Cox regression models for all-cause mortality and cause-specific mortality, showing association with psychological distress (GHQ ≥3).
The 2008 Scania public health survey with 5.3 years follow-up. Stratified by gender: n = 13984 women; 11519 men.

	Model 0		Model 1		Model 2		
**Cause of death**	**HR**	**(95%CI)**	**HR**	**(95%CI)**	**HR**	**(95%CI)**	**Number of deaths**
**All causes**							
Women	**2.1*** **	(1.5-2.8)	1.4	(1.0-1.9)	1.2	(0.9-1.6)	311
Men	**3.6*** **	(2.7-4.7)	**2.8*** **	(2.1-3.8)	**2.4*** **	(1.8-3.2)	464
**Cause-specific: Cardiovascular**							
Women	**2.1* **	(1.0-4.4)	1.2	(0.6-2.5)	1.1	(0.5-2.2)	67
Men	**3.7*** **	(2.4-5.9)	**3.1*** **	(1.8-5.1)	**2.5*** **	(1.6-4.2)	163
**Cancer**							
Women	**1.6* **	(1.0-2.6)	1.2	(0.7-1.9)	1.0	(0.6-1.7)	156
Men	**3.6*** **	(2.4-5.5)	**3.1*** **	(1.9-4.8)	**2.6*** **	(1.7-4.1)	168
**Other causes**							
Women	**2.7* **	(1.4-5.0)	1.7	(1.0-3.1)	1.4	(0.8-2.6)	88
Men	**3.3*** **	(2.0-5.6)	**2.3** **	(1.3-4.0)	**2.0* **	(1.1-3.6)	133

Model 0 adjusted for age.
Model 1 furthermore adjusted for socioeconomic status, physical activity, smoking, and alcohol.
Model 2 furthermore adjusted for chronic disease.
Significance levels: * p<0.05, ** p<0.01, *** p<0.001
Weighted Hazard Ratios. Bootstrap method (2000 replicates) for variation estimation.

For peer review only

In order to **investigate** a dose-response pattern **in the association between GHQ-12 and mortality**, the study population was divided into four groups according to GHQ-score: 0 no distress = reference category (**69** %), 1-2 (**15** %), 3-5 (**8** %), and 6-12 (**8** %). A clear pattern of increasing mortality by increasing distress was seen for men and women combined in **the** fully adjusted cox regression analyses (Table 4). **However, gender-stratification revealed this pattern to be clear and robust among men but not among women.**

For peer review only - http://bmjopen.bmj.com/site/about/guidelines.xhtml

Table 4. **Associations between different levels of psychological distress (GHQ-12) and all-cause mortality.**
The 2008 Scania public health survey with 5.3 years follow-up.
Total population (n=25503). Stratified by gender (n = 13984 women; 11519 men).

GHQ-score	Model 0 HR	Model 0 (95%CI)	Model 1 HR	Model 1 (95%CI)	Model 2 HR	Model 2 (95%CI)	Number of deaths
**Total population**							
0=REF	1.0		1.0		1.0		775
1-2	**1.6*** **	(1.3-2.1)	**1.4***	(1.1-1.7)	1.2	(1.0-1.6)	
3-5	**2.4*****	(1.7-3.2)	**1.8*****	(1.3-2.5)	**1.6****	(1.1-2.2)	
6-12	**3.9*****	(3.0-5.1)	**2.7*****	(2.0-3.6)	**2.2*****	(1.6-3.0)	
**Women**							311
0=REF	1.0		1.0		1.0		
1-2	**1.7*****	(1.2-2.4)	1.3	(0.9-1.9)	1.1	(0.8-1.7)	
3-5	**2.3*****	(1.4-3.6)	**1.6***	(1.0-2.5)	1.4	(0.9-2.2)	
6-12	**2.2*****	(1.5-3.4)	1.3	(0.9-2.1)	1.1	(0.7-1.7)	
**Men**							464
0=REF	1.0		1.0		1.0		
1-2	**1.6****	(1.1-2.2)	**1.4***	(1.0-1.9)	1.3	(1.0-1.8)	
3-5	**2.4*****	(1.6-3.6)	**2.0*****	(1.3-3.1)	**1.7***	(1.1-2.6)	
6-12	**5.2*****	(3.7-7.3)	**3.9*****	(2.7-5.6)	**3.2*****	(2-2-4.7)	

Model 0 adjusted for age (and gender in analysis of total population).
Model 1 furthermore adjusted for socioeconomic status, physical activity, smoking, and alcohol.
Model 2 furthermore adjusted for chronic disease.
Significance levels: * p<0.05, ** p<0.01, *** p<0.001
Weighted Hazard Ratios. Bootstrap method (2000 replicates) for variation estimation.

Figure 1 illustrates the age-adjusted gender difference in all-cause mortality between non-distressed and distressed men and women, respectively. As stated in Table 3, the estimated age-adjusted hazard ratio (HR) was higher for distressed vs. non-distressed men (HR: 3.6; 95% CI: 1.1-4.4) than for distressed vs. non-distressed women (HR: 2.1; 95% CI: 1.5-2.8).

For peer review only - http://bmjopen.bmj.com/site/about/guidelines.xhtml

DISCUSSION

The present study showed a strong and robust association between psychological distress measured by GHQ-12 and 5-year mortality in men, but not in women. Among men, the association held through all adjustments (SES, life-style factors and chronic disease) for all mortality end-points (all-cause mortality and cause-specific mortality; cardiovascular, cancer and other causes). Among women, the initial significant age-adjusted association was lost in the first model (after adjustments for SES and life-style factors) for all mortality end-points. These results are concordant with those of the earlier Finnish longitudinal study on GHQ-12 and 11-year all-cause mortality, i.e. an increased mortality risk mainly explained by excess mortality among distressed men.[9] Earlier research on GHQ-12 and cancer mortality have shown mixed results.[10, 11, 15, 16] In our study the association between psychological distress and cancer mortality held through all adjustments for men and women combined and among men separately.

Among those who died in our study population, more than 50 % reported baseline chronic disease (data not shown). **In line with previous research we found no evidence of reverse causality when adjusting for self-reported chronic disease in the final model for men and women combined,[8, 10, 11, 13, 14] but gender-stratification showed this to be true for men only (the association was non-significant for women earlier in the modelling). The dose-response pattern found between psychological distress (across the full range of severity) and increased risk of all-cause mortality (through all adjustments) for men and women combined was in line with previous research,[8-11] but clear and robust among men only.**

For peer review only - http://bmjopen.bmj.com/site/about/guidelines.xhtml

The gender morbidity-survival paradox was illustrated in this study, as morbidity (psychological distress) was higher among women, while mortality was higher among men. Several potential explanations could be considered. To begin with, the definition of psychological distress could be gendered per se,[22] as expressions of mental ill-health may differ between men and women.[17] Men's and women's overall mental health could be similar, although expressed as different conditions - depression may be thought of as a female problem because more women seek help for depression, but more men commit suicide and turn to drinking and drug-abuse to alleviate symptoms of mental ill-health.[17] The GHQ-12 asks for symptoms of anxiety, depression, sleep disturbance, loss of confidence and the ability to perform daily activities and cope with everyday problems. Men may find it more difficult than women to recognize and report symptoms involving vulnerability because of masculine gender norms,[22] with an ensuing higher threshold to report psychological symptoms on questionnaires. Consequently, the men who do report symptoms might have a more severe disorder as suggested by their heightened mortality risk.[23] Gender is a ubiquitous aspect of mental health, present in contexts ranging from formal diagnostic criteria to the ways individuals label, communicate, and cope with their problems.[22] A more ruminative coping style among women may contribute to an increased vulnerability to depression, but women are also more willing to adopt a sick role, seek help and access health care, which facilitates earlier detection.[20, 24] Clinical depression is about twice as common in women as in men.[24] Sex differences in depression begin during early adolescence and are most pronounced during the reproductive years, and a possible biological explanation to female susceptibility to depression could be associated with estrogen and progesterone.[17, 24] The heritability of depression is also greater in women than men. Sex differences in monoamine functioning have been demonstrated (e.g. a decrease in

For peer review only

serotonin transmission increased depression significantly more in women than men), and women also respond better to antidepressive pharmacological treatment with SSRIs (Selective serotonin reuptake inhibitors) than men.[24] Furthermore, economic explanations and environmental factors may contribute to the gender difference in depression prevalence, such as a female disadvantage of lower socioeconomic status, gender pay gap, strain from dual (work-family) roles and higher rates of exposure to IPV (intimate partner violence).[18, 24]

A recent study investigated psychological distress and heart disease mortality in the United States, with data from more than half a million participants in the National Health Interview Survey (NHIS) and 18 years of mortality follow-up.[25] Results showed a robust dose-response relationship between psychological distress (measured by Kessler 6 item psychological distress scale, K-6) and heart disease mortality, indicating psychological distress to be an important predictor of heart disease mortality. The relative risk of heart disease mortality associated with psychological distress was higher among men than women.[25] Another study highlighted the importance of identifying men with psychological distress when assessing CVD risk, as psychological distress was associated with higher cardiovascular risk scores among men but not women.[26] Psychological distress may increase the risk of heart disease through various mechanisms, e.g. promoting inflammatory processes, and activating automatic nerve system or hypothalamic-pituitary-adrenal (HPA) influences.[25] Psychological distress is also highly correlated with traditional CVD risk factors such as smoking, physical inactivity, obesity, hypertension, and adherence to medical treatment.[12, 25] Further research is needed to address pathways through which psychological distress increases mortality and how different underpinning pathways will be responsible for gendered patterns of morbidity and mortality in different health outcomes.[18, 19, 25] The

For peer review only - http://bmjopen.bmj.com/site/about/guidelines.xhtml

underlying mechanisms in gender differences need to be better understood in order to develop effective public-health and clinical interventions. **Our study supports the validity of GHQ-12 as a predictor of mortality, especially among men. It has been suggested that GHQ-12 might possibly be an even stronger predictor of mortality than CIDI-diagnosed mood/anxiety disorders, since diagnosed mental disorders tend to be treated whereas self-reported psychological distress does not.[11] In the future, it would be valuable to validate screening tools for psychological distress separately for men and women.[24]**

A minor additional aim of the present study was to compare two different GHQ-12 cut-off scores: GHQ-12 ≥3 (conventionally used in Sweden) and GHQ-12 ≥4 (widely used internationally). The choice of a cut-off point depends on the purpose of the study, and a recent Swedish case-control study concluded that although the cut-off points 2-4 were all deemed acceptable, the best sensitivity and specificity of GHQ-12 when discriminating between healthy controls and psychiatric outpatients was seen at cut-off ≥4.[7] GHQ-12 ≥3 defined 17.9 % of our study population as psychologically distressed (women: 20.2 %; men: 15.7 %), while GHQ-12 ≥4 defined 14.2 % as psychologically distressed (women: 16.2 %; men: 12.1 %) (Supplementary Table 1). The higher cut-off led to slightly stronger associations for all mortality end-points except for cancer which showed the same association for men and women combined (Supplementary Table 2), however weaker among women and stronger among men (Supplementary Table 3).

Strengths and limitations

This was a large prospective community study based on a stratified randomized sample of people in Scania 18-80 years old who participated in the 2008 Scania Public Health Survey. **The response rate of 54 % is a limitation, but a weighting variable was specifically**

For peer review only - http://bmjopen.bmj.com/site/about/guidelines.xhtml

designed to compensate for self selection bias in the statistical analyses. Non-responders were more often young, male, low-educated or foreign-born. We do not know if non-responders had more psychological distress than responders, but there were probably more non-responders among persons with serious psychiatric disease (such as psychosis, schizophrenia, severe depression) who are known to have a higher risk of premature death[27] than in the general population, due to the mental task involved in answering a large survey with 134 main questions (totalling 273 items including subqueries and follow-up questions). Most base-line data was self-reported **and our** study **lacked** objective measures of health such as blood pressure, verified physical/ mental illness and biomarkers. We had no objective measure of pre-existing disease at baseline, and self-reported chronic disease may not be a valid indicator of pre-existing disease. The mortality rates across age strata were similar to national data.[28] **P**sychological distress was measured at baseline only, and the persistence of distress may be an important determinant of mortality risk.[29] **With an instrument such as the GHQ-12 asking for symptoms during "the past few weeks" a smaller portion of persons with short-term/ intermittent than long-term/ persistent psychological distress will be identified at baseline. Over time, some of the participants defined as cases at baseline will recover while some defined as non-cases will develop psychological distress, a form of mis-classification diluting the effect of psychological distress on mortality over time (length-time bias).[13, 30, 31] Statistical tests on our study data indicated absence of perfect proportionality with regard to poor GHQ-12 and mortality across the 5.3-year period (see Supplemental file on PH assumption). This is an interesting incidental finding, plausibly explained in part by length time bias and in line with previous research.[13, 25] For example, a large prospective study which investigated the temporal robustness of psychological distress (Kessler 6 items, K-6) by**

For peer review only

For peer review only - http://bmjopen.bmj.com/site/about/guidelines.xhtml

re-estimating the mortality impact at 2-year, 5-year, and 10-year follow-up times, found that the mortality impact of baseline psychological distress was diluted over time.[25]

Conclusions

The present study extends previous research by investigating gender differences in the association between psychological distress measured by GHQ-12 and mortality. Results showed strong and robust associations in men but not in women. GHQ-12 could be used as one of several predictors of mortality, especially among men. The results of the present study are probably mainly generalizable to other western countries, as both exposure and outcome are highly internationally validated measures, but the mediating factors between psychological distress and mortality may be more culturally diverse.

Contributorship statement: ML and MF designed the study in collaboration with MR. ML assembled the database. MP analysed the data with input from ML and MF. MF wrote the first draft. All authors contributed to the paper and approved the final draft.

Competing interests: None declared.

Funding: This work was supported by the Swedish Research Council (Vetenskapsrådet) [K2014-69X-22427-01-4] and the Swedish ALF Government [Grant Dnr F 2014/354].

Data sharing statement: No additional data available.

Acknowledgements: The authors wish to thank all participants for their cooperation, and Region Skåne (Scania) for providing data from the 2008 Scania public health survey.

For peer review only - http://bmjopen.bmj.com/site/about/guidelines.xhtml

For peer review only

REFERENCES

1. Calling S, Midlöv P, Johansson S-E, *et al*. Longitudinal trends in self-reported anxiety. Effects of age and birth cohort during 25 years. *BMC Psychiatry* 2017;17:119. doi: 10.1186/s12888-017-1277-3.

2. Chiu M, Vigod S, Rahman F, *et al*. Mortality risk associated with psychological distress and major depression: A population-based cohort study. *J Affect Disord* 2018;234:117-23. doi: 10.1016/j.jad.2018.02.075.

3. Pedersen SS, von Känel R, Tully PJ, *et al.* Psychosocial perspectives in cardiovascular disease. *Eur J Prev Cardiol* 2017;24(3):108-115. doi: 10.1177/2047487317703827.

4. Bortolato B, Hyphantis TN, Valpione S, *et al.* Depression in cancer: The many biobehavioral pathways driving tumor progression. *Cancer Treat Rev* 2017;52:58-70. doi: 10.1016/j.ctrv.2016.11.004.

5. Goldberg DP, Gater R, Sartorius N, *et al*. The validity of two versions of the GHQ in the WHO study of mental illness in general health care. *Psychol Med* 1997;27:191-7. doi: 10.1017/s0033291796004242.

6. McDowell I. Measuring health: A guide to rating scales and questionnaires. 3rd edition. New York: Oxford University Press 2006:259-72.

For peer review only

For peer review only - http://bmjopen.bmj.com/site/about/guidelines.xhtml

7. Lundin A, Åhs J, Åsbring N, *et al*. Discriminant validity of the 12-item version of the general health questionnaire in a Swedish case-control study. *Nord J Psychiatry* 2017;71:171-9. doi: 10.1080/08039488.2016.1246608.

8. Robinson KL, McBeth J, Macfarlane GJ. Psychological distress and premature mortality in the general population: A prospective study. *Ann Epidemiol* 2004;14:467-72. doi: 10.1016/j.annepidem.2003.11.007.

9. Puustinen PJ, Koponen H, Kautiainen H, *et al*. Psychological distress measured by the GHQ-12 and mortality: A prospective population-based study. *Scand J Public Health* 2011;39(6):577-81. doi: 10.1177/1403494811414244.

10. Russ TC, Stamatakis E, Hamer M, *et al*. Association between psychological distress and mortality: individual participants pooled analysis of 10 prospective cohort studies. *BMJ* 2012;345:e4933. doi: 10.1136/bmj.e4933.

11. Ponizovsky AM, Haklai Z, Goldberger N. Association between psychological distress and mortality: the case of Israel. *J Epidemiol Community Health* 2018;72(8):726-32. doi: 10.1136/jech-2017-210356.

For peer review only

For peer review only - http://bmjopen.bmj.com/site/about/guidelines.xhtml

12. Hamer M, Molloy GJ, Stamatakis E. Psychological distress as a risk factor for cardiovascular events: pathophysiological and behavioral mechanisms. *J Am Coll Cardiol* 2008;52(25):2156-62. doi: 10.1016/j.jacc.2008.08.057.

13. Hamer M, Kivimaki M, Stamatakis E, *et a*l. Psychological distress as a risk factor for death from cerebrovascular disease. *CMAJ* 2012;184(13):1461-6. doi: 10.1503/cmaj.111719.

14. Batty GD, Russ TC, Stamatakis E, *et al*. Psychological distress and risk of peripheral vascular disease, abdominal aortic aneurysm, and heart failure: Pooling of sixteen cohort studies. *Atherosclerosis* 2014;236(2):385-8. doi: 10.1016/j.atherosclerosis.2014.06.025.

15. Hamer M, Chida Y, Molloy GJ. Psychological distress and cancer mortality. *J Psychosom Res* 2009;66(3):255-8. doi: 10.1016/j.jpsychores.2008.11.002.

16. Batty GD, Russ TC, Stamatakis E, *et al*. Psychological distress in relation to site specific cancer mortality: pooling of unpublished data from 16 prospective cohort studies. *BMJ* 2017;356:j108. doi: 10.1136/bmj.j108.

17. Rieker PP, Bird CE. Rethinking gender differences in health: Why we need to integrate social and biological perspectives. *J Gerontol B Psychol Sci Soc Sci* 2005;60(2):4-7. doi: 10.1093/geronb/60.special_issue_2.s40.

For peer review only

18. Bambra C, Albani V, Franklin P. COVID-19 and the gender health paradox. *Scand J Public Health* 2021;49(1):17-26. doi: 10.1177/1403494820975604.

19. Oksuzyan A, Brønnum-Hansen H, Jeune B. Gender gap in health expectancy. *Eur J Ageing* 2010;7(4):213-8. doi: 10.1007/s10433-010-0170-4.

20. Gordon EH, Hubbard RE. Do sex differences in chronic disease underpin the sex-frality paradox? *Mech Ageing Dev* 2019;179:44-50. doi: 10.1016/j.mad.2019.02.004.

21. SAS/STAT Software Survey Analysis https://support.sas.com/rnd/app/stat/procedures/SurveyAnalysis.html (accessed 31 July 2021).

22. Addis ME, Hoffman E. Men's depression and help-seeking through the lenses of gender. In: Levant RF, Wong YJ, eds. The Psychology of Men and Masculinities. American Psychological Association 2017:171-196.

23. Gunnell D, Rasul F, Stansfeld SA, *et al*. Gender differences in self-reported minor mental disorder and its association with suicide. A 20-year follow-up of the Renfrew and Paisley cohort. *Soc Psychiatry Psychiatr Epidemiol* 2002;37(10):457–9. doi: 10.1007/s00127-002-0579-y.

24. Shah BM, Kornstein SG. Mental health: Sex and gender evidence in depression, general anxiety disorder, and schizophrenia. In: Jenkins MR, Newman CB, eds. How

For peer review only - http://bmjopen.bmj.com/site/about/guidelines.xhtml

sex and gender impact clinical practice. An evidence-based guide to patient care. Elsevier and Academic Press 2021:153-169.

25. Lee H, Singh GK. Psychological distress and heart disease mortality in the United States: Results from the 1997-2014 NHIS-NDI Record Linkage Study. ***Int J MCH AIDS*** **2020;9(3):260-73.** doi: 10.21106/ijma.391.

26. Puustinen PJ, Koponen H, Kautiainen H, ***et al*****. Gender-specific association of psychological distress with cardiovascular risk scores.** ***Scand J Prim Health Care*** **2010; 28(1):36-40. doi: 10.3109/02813431003648131.**

27. Ösby U, Westman J, Hällgren J, *et al*. Mortality trends in cardiovascular causes in schizophrenia, bipolar and unipolar mood disorder in Sweden 1987-2010. *Eur J Public Health* 2016;26(5):867-71. doi: 10.1093/eurpub/ckv245.

28. The Public Health Agency (Folkhälsomyndigheten). Public health in Sweden 2016. Annual report. (Folkhälsan i Sverige 2016. Årlig rapportering.) In Swedish. Available from: https://www.folkhalsomyndigheten.se/contentassets/cc89748e004743c39ff4c03fec24c570/folkhalsan-i-sverige-2016-16005.pdf (accessed 14 July 2021).

29. Stewart RAH, Colquhoun DM, Marschner SL, *et al*. Persistent psychological distress and mortality in patients with stable coronary artery disease. *Heart* 2017;103(23):1860-6. doi: 10.1136/heartjnl-2016-311097.

For peer review only

30. Rothman, K. Epidemiology. An Introduction. Oxford, New York: Oxford University Press 2002:204.

31. Aschengrau A, Seage III GR. Epidemiology in Public Health. Third Edition. Burlington, MA: Jones and Bartlett Learning 2014:442.

Figure 1. Age-adjusted survival curves of all-cause mortality among non-distressed (GHQ-12 <3) and distressed (GHQ-12 ≥3) participants, divided by gender.

The 2008 Scania public health survey with 5.3 years follow-up. N=11519 men and 13984 women. Weighted data.

For peer review only - http://bmjopen.bmj.com/site/about/guidelines.xhtml

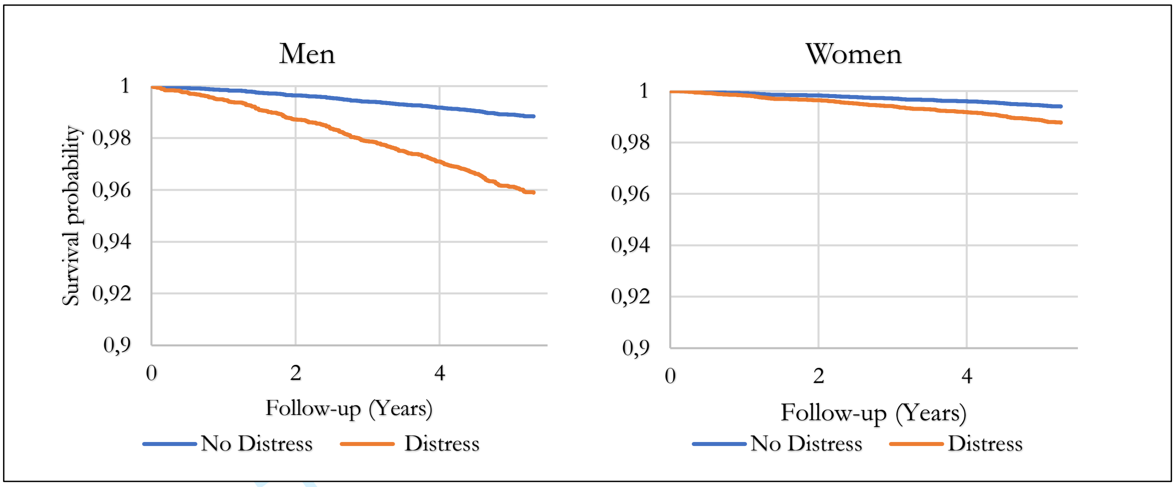

Figure 1. Age-adjusted survival curves of all-cause mortality among non-distressed (GHQ-12 < 3) and distressed (GHQ-12 ≥ 3) participants, divided by gender.

The 2008 Scania public health survey with 5.3 years follow-up. N=11519 men and 13984 women. Weighted data.

For peer review only

Supplementary Table 1. Descriptive characteristics (%) of age, SES, sedentary leisure time, daily smoking, alcohol, BMI, and chronic distress by psychological distress (GHQ-12≥4).
The 2008 Public Health Survey of Scania, Sweden. Total population n=25503. Weighted prevalence.

	Women			Men		
	n = 13984			n = 11519		
	Psychological distress			Psychological distress		
	**No**	**Yes**	p-value	**No**	**Yes**	p-value
	n = 11950	n = 2034		n = 10283	n = 1236	
	83.8 %	**16.2 %**		87.9 %	**12.1 %**	
**Age**, yrs: mean ± SD [a]	46.6 ± 16.0 (46.2-47.0)	40.6 ± 16.6 (39.7-41.4)	<0.001	47.2 ± 17.7 (46.8-47.6)	44.0± 17.5 (42.9-45.0)	<0.001
**Body Mass Index (BMI)**: mean ± SD [a]	24.9 ± 4.6 (24.8-25.1)	24.9 ± 5.3 (24.7-25.2)	0.931	26.1 ± 4.1 (26.0-26.2)	26.4 ± 5.1 (26.0-26.7)	0.102
**Socioeconomic status (SES)** [b]						
Higher non-manual	7.9 (7.3-8.4)	6.8 (5.5-8.1)		9.8 (9.1-10.5)	9.4 (7.5-11.3)	
Medium non-manual	15.7 (15.0-16.5)	13.8 (12.0-15.6)		12.3 (11.5-13.1)	8.5 (6.7-10.3)	
Lower non-manual	10.6 (9.9-11.3)	9.6 (8.1-11.2)		5.3 (4.8-5.9)	5.1 (3.6-6.6)	
Skilled manual	9.5 (8.6-10.1)	7.6 (6.1-9.0)		12.0 (11.2-12.8)	10.1 (7.9-12.3)	
Unskilled manual	12.6 (11.8-13.3)	10.5 (8.9-12.1)		13.4 (12.5-14.2)	9.6 (7.5-11.6)	
Self-employed/farmer	4.1 (3.7-4.6)	3.1 (2.1-4.1)		8.3 (7.6-9.0)	6.4 (4.8-8.0)	
Early retired	3.9 (3.5-4.4)	7.3 (5.8-8.7)		2.6 (2.2-3.0)	10.1 (7.7-12.4)	
Unemployed	3.3 (2.8-3.7)	9.3 (7.6-11.0)		3.0 (2.5-3.5)	10.1 (7.9-12.4)	
Student	8.8 (8.1-9.5)	13.2 (11.1-15.3)		6.5 (5.9-7.2)	9.9 (7.5-12.3)	
Old age pensioner	18.6 (17.8-19.4)	9.9 (8.4-11.3)		19.9 (19.0-20.8)	10.1 (8.4-11.9)	
Unclassified	4.2 (3.7-4.7)	4.5 (3.3-5.7)		6.4 (5.7-7.1)	6.6 (4.6-8.5)	
Long-term sickleave	0.8 (0.6-1.0)	4.4 (3.3-5.6)	<0.001	0.5 (0.3-0.6)	4.2 (2.9-5.5)	<0.001
**Sedentary leisure time** [b]	11.2 (10.4-11.9)	21.4 (19.1-23.7)	<0.001	13.7 (12.8-14.6)	28.9 (25.6-32.1)	<0.001
**Daily smoking** [b]	14.7 (13.9–15.5)	19.6 (17.5-21.8)	<0.001	12.7 (11.8-13.5)	22.1 (19.1-25.1)	<0.001
**Alcohol drinking past year**[b]						
Never	14.2 (13.3-15.0)	18.6 (16.5-20.8)		8.6 (7.9-9.4)	14.8 (12.1-17.5)	
Once a month or more seldom	27.1 (26.1-28.1)	28.6 (26.1-31.0)		18.2 (17.3-19.2)	23.4 (20.4-26.5)	
2-4 times a month	34.6 (33.5-35.6)	31.5 (28.9-34.0)		37.8 (36.7-39.0)	29.5 (26.3-32.6)	
2-3 times a week	19.7 (18.9-20.6)	15.3 (13.5-17.2)		25.8 (24.8-26.8)	22.5 (19.6-25.4)	
At least 4 times a week	4.4 (4.0-4.9)	6.0 (4.8-7.1)	<0.001	9.4 (8.8-10.1)	9.9 (8.0-11.7)	<0.001
**Chronic disease*** [b]	26.8 (25.9-27.8)	45.3 (42.7-48.0)	<0.001	24.8 (23.8-25.8)	47.8 (44.4-51.2)	<0.001

GHQ-12 = 12 item version of the General Health Questionnaire, 0-12 points. Psychological distress was defined as GHQ-12≥4.
* Chronic disease = long-term disease, injury-related trouble, disability or other weakness.
[a] p-value: Independent samples T-test, 2-tailed
[b] p-value: Pearson Chi Square test, 2-sided
The values in parentheses are 95% confidence intervals for mean or percent based on bootstrap method with 2000 number of replicates.

For peer review only

Supplementary Table 2. HRs from Cox regression models for all-cause mortality and cause-specific mortality, showing association with psychological distress (GHQ≥4).
The 2008 Scania public health survey with 5.3 years follow-up. Men and women combined, n=25503.

	Model 0		Model 1		Model 2		
**Cause of death**	**HR**	**(95%CI)**	**HR**	**(95%CI)**	**HR**	**(95%CI)**	**Number of deaths**
**All causes**	**3.2***	(2.5-4.0)	**2.3***	(1.8-3.0)	**2.0***	(1.5-2.5)	775
**Cause-specific:**							
**Cardiovascular**	**3.6***	(2.4-5.4)	**2.5***	(1.6-4.0)	**2.2***	(1.4-3.4)	230
**Cancer**	**2.6***	(1.8-3.8)	**2.0***	(1.4-2.9)	**1.7***	(1.2-2.5)	324
**Other causes**	**3.6***	(2.4-5.5)	**2.4***	(1.5-3.6)	**2.0**	(1.3-3.2)	221

Model 0 adjusted for age and gender.
Model 1 furthermore adjusted for socioeconomic status, physical activity, smoking, and alcohol.
Model 2 furthermore adjusted for chronic disease.
Significance levels: * p<0.05, ** p<0.01, *** p<0.001
Weighted Hazard Ratios. Bootstrap method (2000 replicates) for variation estimation.

For peer review only

Supplementary Table 3. HRs from Cox regression models for all-cause mortality and cause-specific mortality, showing association with psychological distress (GHQ≥4).
The 2008 Scania public health survey with 5.3 years follow-up. Stratified by gender: n = 13984 women; 11519 men.

	Model 0		Model 1		Model 2		
**Cause of death**	**HR**	**(95%CI)**	**HR**	**(95%CI)**	**HR**	**(95%CI)**	**Number of deaths**
**All causes**							
Women	**2.3*** **	(1.7-3.3)	**1.5***	(1.0-2.1)	1.3	(0.9-1.8)	311
Men	**3.9*** **	(2.9-5.2)	**3.0*** **	(2.2-4.1)	**2.5*** **	(1.9-3.5)	464
**Cause-specific:**							
**Cardiovascular**							
Women	**2.8** **	(1.3-5.8)	1.5	(0.7-3.1)	1.4	(0.7-2.8)	67
Men	**3.9*** **	(2.4-6.4)	**3.1*** **	(1.8-5.5)	**2.7*** **	(1.6-4.5)	163
**Cancer**							
Women	1.5	(0.9-2.6)	1.0	(0.6-1.8)	0.9	(0.5-1.6)	156
Men	**3.9*** **	(2.5-6.1)	**3.2*** **	(2.0-5.3)	**2.8*** **	(1.8-4.4)	168
**Other causes**							
Women	**3.4*** **	(1.8-6.3)	**2.1** **	(1.1-3.8)	1.8	(1.0-3.2)	88
Men	**3.7*** **	(2.1-6.6)	**2.5*** **	(1.4-4.6)	**2.2***	(1.2-4.2)	133

Model 0 adjusted for age.
Model 1 furthermore adjusted for socioeconomic status, physical activity, smoking, and alcohol.
Model 2 furthermore adjusted for chronic disease.
Significance levels: * p<0.05, ** p<0.01, *** p<0.001
Weighted Hazard Ratios. Bootstrap method (2000 replicates) for variation estimation.

For peer review only

Supplementary Table 4. Associations between different levels of psychological distress (GHQ-12) and all-cause mortality.
The 2008 Scania public health survey with 5.3 years follow-up.
Total population (n=25503). Stratified by gender (n = 13984 women; 11519 men).

GHQ-score	Model 0 HR	Model 0 (95%CI)	Model 1 HR	Model 1 (95%CI)	Model 2 HR	Model 2 (95%CI)	Number of deaths
**Total population**							
0=REF	1.0		1.0		1.0		775
1-3	**1.7*** **	(1.3-2.1)	**1.4** **	(1.1-1.8)	1.2	(1.0-1.6)	
4-6	**2.8*** **	(2.0-3.9)	**2.1*** **	(1.5-2.9)	**1.8*** **	(1.3-2.5)	
7-12	**4.1*** **	(3.1-5.6)	**2.8*** **	(2.0-3.9)	**2.3*** **	(1.7-3.2)	
**Women**							
0=REF							311
1-3	**1.6** **	(1.1-2.2)	1.2	(0.8-1.7)	1.1	(0.8-1.6)	
4-6	**2.9*** **	(1.8-4.7)	**1.9** **	(1.1-3.0)	1.6	(1.0-2.6)	
7-12	**2.3*** **	(1.5-3.6)	1.3	(0.8-2.1)	1.1	(0.7-1.8)	
**Men**							
0=REF							464
1-3	**1.7*** **	(1.3-2.3)	**1.5** **	(1.1-2.0)	1.3	(1.0-1.8)	
4-6	**2.8*** **	(1.8-4.3)	**2.2** **	(1.3-3.5)	**1.9*** **	(1.2-3.0)	
7-12	**5.5*** **	(3.8-8.0)	**4.2*** **	(2.8-6.2)	**3.5*** **	(2.3-5.2)	

Model 0 adjusted for age (and gender in analysis of total population).
Model 1 furthermore adjusted for socioeconomic status, physical activity, smoking, and alcohol.
Model 2 furthermore adjusted for chronic disease.
Significance levels: * p<0.05, ** p<0.01, *** p<0.001
Weighted Hazard Ratios. Bootstrap method (2000 replicates) for variation estimation.
GHQ-score total population: 0 no distress = reference category (66 %), 1-3 (20 %), 4-6 (7 %), and 7-12 (7 %).

For peer review only

Tests assessing the proportional hazards assumption

The proportional hazards assumption was considered fulfilled after inspection of the survival curves according to psychological distress (yes/no). However, statistical tests on our study data indicated absence of perfect proportionality regarding poor GHQ-12 and mortality across the 5.3-year period (see below). This finding is in line with results of other studies showing a dilution over time for the mortality impact of baseline psychological distress.

1. Plots of log (- log S(t))

The two curves are not perfectly parallel in the Log-Negative Log of Estimated Survivor Functions by psychological distress.

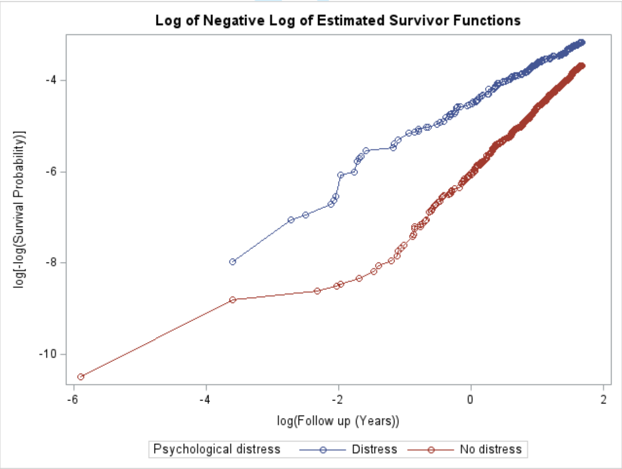

.

2. Interactions with Time

We used a product term between time and psychological distress to test the proportionality assumption. The interaction term with chi-square (X^2), *1 df* ($X^2 = 37.2$, *P <.001*) shows that this effect is statistically significant in the fitted model.

3. Schoenfeld residuals

The Schoenfeld residuals plot for psychological distress suggests that the coefficient for psychological distress changes over time, i.e: the mortality impact is diluted over time.

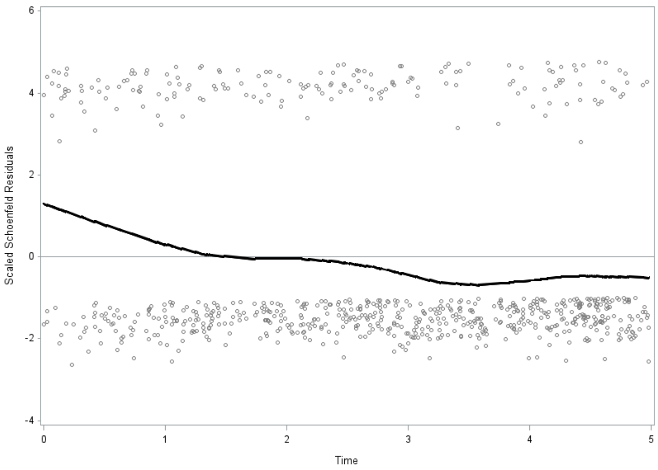

For peer review only - http://bmjopen.bmj.com/site/about/guidelines.xhtml

STROBE Statement—Checklist of items that should be included in reports of ***cohort studies***

	Item No	Recommendation	Page No
**Title and abstract**	1	(*a*) Indicate the study's design with a commonly used term in the title or the abstract	Title
		(*b*) Provide in the abstract an informative and balanced summary of what was done and what was found	Abstract
**Introduction**			
Background/rationale	2	Explain the scientific background and rationale for the investigation being reported	3-6
Objectives	3	State specific objectives, including any prespecified hypotheses	6
**Methods**			
Study design	4	Present key elements of study design early in the paper	7-8
Setting	5	Describe the setting, locations, and relevant dates, including periods of recruitment, exposure, follow-up, and data collection	7
Participants	6	(*a*) Give the eligibility criteria, and the sources and methods of selection of participants. Describe methods of follow-up	7-8
		(*b*) For matched studies, give matching criteria and number of exposed and unexposed	
Variables	7	Clearly define all outcomes, exposures, predictors, potential confounders, and effect modifiers. Give diagnostic criteria, if applicable	8-9
Data sources/ measurement	8*	For each variable of interest, give sources of data and details of methods of assessment (measurement). Describe comparability of assessment methods if there is more than one group	8-9
Bias	9	Describe any efforts to address potential sources of bias	7
Study size	10	Explain how the study size was arrived at	7
Quantitative variables	11	Explain how quantitative variables were handled in the analyses. If applicable, describe which groupings were chosen and why	8-9
Statistical methods	12	(*a*) Describe all statistical methods, including those used to control for confounding	9-11
		(*b*) Describe any methods used to examine subgroups and interactions	10
		(*c*) Explain how missing data were addressed	7
		(*d*) If applicable, explain how loss to follow-up was addressed	7
		(*e*) Describe any sensitivity analyses	
**Results**			
Participants	13*	(a) Report numbers of individuals at each stage of study—eg numbers potentially eligible, examined for eligibility, confirmed eligible, included in the study, completing follow-up, and analysed	7
		(b) Give reasons for non-participation at each stage	7
		(c) Consider use of a flow diagram	
Descriptive data	14*	(a) Give characteristics of study participants (eg demographic, clinical, social) and information on exposures and potential confounders	Table 1 page 12
		(b) Indicate number of participants with missing data for each variable of interest	
		(c) Summarise follow-up time (eg, average and total amount)	8
Outcome data	15*	Report numbers of outcome events or summary measures over time	Abstract Tables 2-4

For peer review only - http://bmjopen.bmj.com/site/about/guidelines.xhtml

Main results	16	(*a*) Give unadjusted estimates and, if applicable, confounder-adjusted estimates and their precision (eg, 95% confidence interval). Make clear which confounders were adjusted for and why they were included<br>(*b*) Report category boundaries when continuous variables were categorized<br>(*c*) If relevant, consider translating estimates of relative risk into absolute risk for a meaningful time period	Table 2-4 p14-16
Other analyses	17	Report other analyses done—eg analyses of subgroups and interactions, and sensitivity analyses	10
**Discussion**			
Key results	18	Summarise key results with reference to study objectives	17
Limitations	19	Discuss limitations of the study, taking into account sources of potential bias or imprecision. Discuss both direction and magnitude of any potential bias	20
Interpretation	20	Give a cautious overall interpretation of results considering objectives, limitations, multiplicity of analyses, results from similar studies, and other relevant evidence	22
Generalisability	21	Discuss the generalisability (external validity) of the study results	22
**Other information**			
Funding	22	Give the source of funding and the role of the funders for the present study and, if applicable, for the original study on which the present article is based	22

*Give information separately for exposed and unexposed groups.

Note: An Explanation and Elaboration article discusses each checklist item and gives methodological background and published examples of transparent reporting. The STROBE checklist is best used in conjunction with this article (freely available on the Web sites of PLoS Medicine at http://www.plosmedicine.org/, Annals of Internal Medicine at http://www.annals.org/, and Epidemiology at http://www.epidem.com/). Information on the STROBE Initiative is available at http://www.strobe-statement.org.

For peer review only

BMJ Open

Poor psychological health and 8-year mortality: A population-based prospective cohort study stratified by gender in Scania, Sweden

Journal:	*BMJ Open*
Manuscript ID	bmjopen-2021-056367.R1
Article Type:	Original research
Date Submitted by the Author:	25-Feb-2022
Complete List of Authors:	Fridh, Maria; Lund University Faculty of Medicine, Social Medicine and Health Policy, Department of Clinical Sciences in Malmö<br>Pirouzifard, Mirnabi; Lund University Faculty of Medicine, Social Medicine and Health Policy, Department of Clinical Sciences in Malmö; Region Skåne and Lund University, Centre for Primary Health Care Research<br>Rosvall, Maria; Lund University Faculty of Medicine, Social Medicine and Health Policy, Department of Clinical Sciences in Malmö; University of Gothenburg Sahlgrenska Academy, School of Public Health, Institute of Medicine<br>Lindstrom, Martin; Lund University Faculty of Medicine, Social Medicine and Health Policy, Department of Clinical Sciences in Malmö; Region Skåne and Lund University, Centre for Primary Health Care Research
**Primary Subject Heading**:	Epidemiology
Secondary Subject Heading:	Mental health, Public health
Keywords:	PUBLIC HEALTH, EPIDEMIOLOGY, MENTAL HEALTH, SOCIAL MEDICINE

SCHOLARONE™
Manuscripts

I, the Submitting Author has the right to grant and does grant on behalf of all authors of the Work (as defined in the below author licence), an exclusive licence and/or a non-exclusive licence for contributions from authors who are: i) UK Crown employees; ii) where BMJ has agreed a CC-BY licence shall apply, and/or iii) in accordance with the terms applicable for US Federal Government officers or employees acting as part of their official duties; on a worldwide, perpetual, irrevocable, royalty-free basis to BMJ Publishing Group Ltd ("BMJ") its licensees and where the relevant Journal is co-owned by BMJ to the co-owners of the Journal, to publish the Work in this journal and any other BMJ products and to exploit all rights, as set out in our licence.

The Submitting Author accepts and understands that any supply made under these terms is made by BMJ to the Submitting Author unless you are acting as an employee on behalf of your employer or a postgraduate student of an affiliated institution which is paying any applicable article publishing charge ("APC") for Open Access articles. Where the Submitting Author wishes to make the Work available on an Open Access basis (and intends to pay the relevant APC), the terms of reuse of such Open Access shall be governed by a Creative Commons licence – details of these licences and which Creative Commons licence will apply to this Work are set out in our licence referred to above.

Other than as permitted in any relevant BMJ Author's Self Archiving Policies, I confirm this Work has not been accepted for publication elsewhere, is not being considered for publication elsewhere and does not duplicate material already published. I confirm all authors consent to publication of this Work and authorise the granting of this licence.

For peer review only

Poor psychological health and 8-year mortality: A population-based prospective cohort study stratified by gender in Scania, Sweden

Maria Fridh[1]*, MD, PhD, Mirnabi Pirouzifard [1,2], PhD, Maria Rosvall[1,3,4], MD, PhD, and Martin Lindström[1,2], MD, PhD

[1] Social Medicine and Health Policy, Department of Clinical Sciences in Malmö, Lund University, CRC, Jan Waldenströmsgata 35, Skåne University Hospital Malmö, SE-205 02 Malmö, Sweden.

[2] Centre for Primary Health Care Research, Region Skåne and Lund University, S-205 02 Malmö, Sweden.

[3] School of Public Health, Institute of Medicine, Sahlgrenska academy, University of Gothenburg, Sweden.

[4] Socialmedicinskt centrum, Regionhälsan, Västra Götalandsregionen, Sweden.

E-mail:

Maria Fridh,* maria.fridh@med.lu.se

Mirnabi Pirouzifard, mirnabi.pirouzifard@med.lu.se

Maria Rosvall, maria.rosvall@gu.se

Martin Lindström, martin.lindstrom@med.lu.se

* Corresponding author

WORD COUNT: 3944

For peer review only - http://bmjopen.bmj.com/site/about/guidelines.xhtml

ABSTRACT

Objectives: We investigated gender differences in the association between mortality and general psychological distress (measured by GHQ-12), as an increased mortality risk has been shown in community studies, but gender differences are largely unknown. **Setting**: We used data from a cross-sectional population-based public health survey conducted in 2008 in the Swedish region of Skåne (Scania) of people 18-80 years old (response rate 54.1 %). The relationship between psychological distress and subsequent all-cause and cause-specific mortality was examined by logistic regression models for the total study population and stratified by gender, adjusting for age, socioeconomic status, life-style (physical activity, smoking, alcohol consumption) and chronic disease. **Participants**: Of 28198 respondents, 25503 were included in analysis by restrictive criteria. **Outcome measures**: Overall and cause-specific mortality by December 31st, 2016. **Results**: More women (20.2 %) than men (15.7 %) reported psychological distress at baseline (GHQ ≥3). During a mean follow-up of 8.1 years, 1389 participants died: 425 (30.6%) from cardiovascular diseases, 539 (38.8%) from cancer, and 425 (30.6%) from other causes. The overall association between psychological distress and mortality risk held for all mortality end-points except cancer after multiple adjustments (e.g. all-cause mortality OR: 1.8 (95 % CI: 1.4 – 2.2) for men and women combined. However, stratification revealed a clear gender difference as the association between GHQ-12 and mortality was consistently stronger and more robust among men than women. **Conclusion:** More women than men reported psychological distress while mortality was higher among men (i.e. the morbidity-mortality gender paradox). GHQ-12 could potentially be used as one of several predictors of mortality, especially for men. In the future, screening tools for psychological distress should be validated for both men and women. Further research regarding the underlying mechanisms of the gender paradox is warranted.

For peer review only - http://bmjopen.bmj.com/site/about/guidelines.xhtml

KEY WORDS: Mortality, Psychological Distress, Prospective Study, Sweden, GHQ-12, Gender Paradox

Strengths and limitations of this study

- This was a large, prospective, randomly selected stratified population-based study with valid exposure and outcome measures (GHQ-12 and mortality).
- The participant rate was moderately high with no substantial selection bias.
- Relevant potential confounders (age, SES, physical activity, smoking, alcohol consumption) were included in the multiple statistical models.
- In order to investigate reverse causality, the final model adjusted for self-reported chronic disease, which may be a less valid indicator than objective measures of pre-existing disease at baseline.
- Although statistical models included several potentially relevant confounders, residual confounding cannot be excluded.

INTRODUCTION

Increasing psychological distress in the general population, especially among young people, is of great public health concern in Sweden.[1] Unspecified psychological distress has been shown to have a high and persistent mortality effect in the general population independently of clinical depression and other severe mental illness.[2] The mechanisms behind this are probably both biological and behavioural with intricate interactions across pathways, contributing to e.g. cardiovascular disease, cancer progression and premature mortality.[3, 4]

The General Health Questionnaire is an extensively used, reliable and validated measure of unspecific psychological distress in population studies,[5, 6] and in Sweden the 12-item

For peer review only - http://bmjopen.bmj.com/site/about/guidelines.xhtml

General Health Questionnaire (GHQ-12) has been used for decades in national and regional surveys.[7] Earlier population-based research has demonstrated an association between psychological distress measured by the GHQ-12 and risk of all-cause mortality,[8-11] as well as cause-specific mortality (e.g. cardiovascular death,[8, 10-14] cancer,[10, 15, 16] and other causes.[11]) The association with GHQ-12 is clear and robust for all-cause mortality and cardiovascular mortality, but mixed for cancer mortality (some studies show an association with higher levels of psychological distress for all-cancer mortality,[10] some for certain types of cancer only,[15, 16] and other studies no association at all.[11]) One confounder of the relationship between psychological distress and premature death might be the presence of pre-existing disease at baseline (contributing both to distress and subsequent higher mortality rates, i.e. "reverse causality").[10] Several studies on GHQ-12 and mortality attempted to evaluate the possibility of reverse causality by adjusting for different measures of baseline physical illness, and/or excluding all deaths the first years of follow-up. Reverse causality was not found to explain the association between GHQ-12 and all-cause mortality or cardiovascular mortality,[8, 10, 11, 13, 14] but results were less clear for cancer mortality.[10, 15, 16] A dose-response relationship between psychological distress and increased risk of mortality may support the existence of a causal relationship,[8] and several studies reported a dose-response pattern for all-cause mortality and cardiovascular mortality [8-11, 13, 14] (some very large studies showed an increased mortality risk even at lower levels of distress).[10, 14] Results on dose-response for cancer mortality were less consistent.[10, 16]

The fact that psychological distress is more often reported by women than men while mortality rates are higher among men is an example of the male-female health-survival paradox.[17-19] Women are in general more likely to acquire disabling conditions such as arthritis, autoimmune diseases and depression, while men are more likely to acquire lethal

For peer review only - http://bmjopen.bmj.com/site/about/guidelines.xhtml

conditions at younger ages, such as lung cancer and myocardial infarction.[17-19] There are multiple explanations for the gender paradox, but it is thought that both sex (biological factors) and gender (social factors) play important and interacting roles.[18] The most prominent biological explanations are *genetic* (two X-chromosomes constitute a survival/ageing advantage), *hormonal* (e.g. the protective effect of estrogens on serum lipids delaying atherosclerosis in females), and *immunological* (testosterone may reduce the robustness of the male immunological system with a higher risk of life-threatening infections, while immunological effects of estrogens may increase the risk of autoimmunity and chronic inflammation).[19, 20] Social explanations focus on differences in *behavior and social roles*. Men are more likely than women to engage in health damaging risk-taking behaviours such as excessive alcohol consumption, smoking, drug use and unsafe driving. Gender roles may also contribute to differences in willingness and ability to adopt a sick role, seek help and access health care (men are more likely to present late with symptoms).[18-20] Despite decades of research, it is still not fully understood whether behavioural factors explain most of the gender gap or whether biological and social differences contribute more substantially to the gender gap in health and mortality.[19]

Gender differences in the association between psychological distress measured by GHQ-12 and mortality have rarely been investigated. In our literature search we found only one study that specifically aimed to examine gender-specific associations between GHQ-12 and all-cause mortality.[9] In this study from Finland, 923 persons (414 men and 509 women) answered GHQ-12 at baseline and were followed during a mean observation time of 11 years with 44 death events (27 men, 17 women). Adjustments were made for gender, age, socioeconomic status, BMI, smoking and physical activity. The hazard ratio (HR) increased for every GHQ-12 point. The 10-year survival for distressed (GHQ $\geq$4) vs non-distressed

For peer review only - http://bmjopen.bmj.com/site/about/guidelines.xhtml

(GHQ <4) men was significantly shorter (HR=3.38 (95%CI: 1.55-7.39) but there was no significant difference in survival between distressed and non-distressed women. The authors concluded that the increased all-cause mortality risk among the psychologically distressed was mainly due to excess mortality among distressed men. However, a weaker significant association among women may not have been detected due to lack of statistical power.

Using a large representative population from the Swedish region of Skåne (Scania) with baseline GHQ-12 (survey data) and 8-year prospective mortality data, the present study aimed to:

1) investigate the association between psychological distress and mortality (all-cause and cause-specific) including adjustment for self-reported chronic disease as an indicator of potential reverse causality; and

2) explore a dose-response effect of psychological distress on all-cause mortality.

Furthermore, as an additional minor aim we wanted to compare two different GHQ-12 cut-off scores: GHQ ≥3 (conventionally used in Swedish public health surveys) [7] and GHQ ≥4 (widely used internationally).[6, 12, 15] All analyses were performed on men and women combined and furthermore stratified by gender.

METHODS

Participants and study design

Data from the cross-sectional 2008 Scania public health survey was used. A total of 52142 persons aged 18–80 years (a random stratified sample selected from the official population registers of people living in Scania including 5.8% of the total population 18-80 years old) were invited to participate. The first information letter was sent out on August 27, 2008 with the option of answering the survey online. The paper questionnaires were sent by regular mail

For peer review only - http://bmjopen.bmj.com/site/about/guidelines.xhtml

on September 5, as well as three reminders. A total of 28198 persons returned completed questionnaires (response rate 54.1 %). Comparison between participants with register statistics of the total population in Scania showed that the age interval 18-34 years was somewhat under-represented and the age interval 65-80 years somewhat over-represented. Some under-representation of men and persons with low formal education was also observed, but the most serious under-representation concerned people not born in Europe. To compensate for selection bias, the geographically stratified random sample was weighted by age, sex, country of birth, marital status, income, and education through a weighting variable designed by Statistics Sweden (SCB). We linked study participants to mortality data and followed them until December 31st, 2016. Of the 28198 respondents in the survey, 136 could not be traced, leaving a cohort of 28062 persons. The study sample was restricted to those with no missing values in covariates used, i.e.; 25503 individuals, 11519 men (45%) and 13984 women (55%). This study was approved by the regional Ethical Committee in Lund, Sweden (No. 2010/343).

Patient and Public Involvement statement (PPI)

The present study was based on data from a community population survey in the Swedish region of Scania. The public was not involved in the design of this study. As the Swedish person numbers were not included in the data delivery, participants cannot be individually identified. Results of the present study will be disseminated to the public through publication in scientific journals.

Predictor and outcome variables

Psychological distress was assessed by the 12-item General Health Questionnaire (GHQ-12).[5, 6] The twelve items reflect different aspects of psychological health such as anxiety,

For peer review only - http://bmjopen.bmj.com/site/about/guidelines.xhtml

depression, sleep disturbance, loss of confidence and the ability to perform daily activities and cope with everyday problems during a time-frame of ”the past few weeks”. Cronbach coefficient alpha showed good internal consistency (men: 0.893; women: 0.896). Interpretation of answers is based on a 4-point response scale scored using a bimodal method (symptom present: not at all = 0, same as usual = 0, more than usual = 1, much more than usual = 1) resulting in a GHQ-score of 0-12. Mean GHQ-score was 1.49 for men and 1.61 for women ($p<0.001$). We used the Swedish conventional score of ≥3 to define psychological distress.[7] To determine whether there was a dose-response relation, we categorized the results from the GHQ-12 into four groups (no distress = 0; sub-clinical distress = 1-2; moderate distress = 3-5; and high distress = 6-12).

Mortality was assessed from August 27, 2008 to December 31, 2016, which amounts to a maximum period of 8.34 years. (Mean value men: 8.01 years; women: 8.13 years. Median value men: 8.28 and women: 8.29 years). Cause of death was assessed according to ICD-10 and subdivided into three mortality categories: 1) cardiovascular causes of death (e.g. myocardial infarction and stroke, ICD I109-I729), 2) death from cancer (C019-C979) and 3) death from other causes (A047-B999, D329-G931, J101-/869). Information regarding mortality and cause of death was obtained from The National Cause of Death Register (Dödsorsaksregistret, Socialstyrelsen).

Covariates

The following covariates were chosen as they are both known predictors of mortality and associated with psychological distress. *Age* was used as a continuous variable. *Socioeconomic status* (SES) was defined by 12 categories of employment: higher non-manual; medium non-manual; lower non-manual; skilled manual; unskilled manual; self-employed/farmer; early

For peer review only - http://bmjopen.bmj.com/site/about/guidelines.xhtml

retired (before age 65 years, for health reasons or entitlement); unemployed; student, old-age pensioner (≥ 65 years of age); unclassified; and long-term sick-leave. The participants' age and SES were registry data from Statistics Sweden (SCB) while all other covariates were self-reported data from the Scania public health survey. Leisure-time *physical activity* was assessed from four alternatives, of which the lowest level, sedentary (walking, bicycling etc. less than 2 hours per week) was dichotomized as "yes" and all other options as "no". Smoking was assessed by three categories: daily; intermittent/ non-daily; non-smoker, of which *daily smoking* was dichotomized as "yes" and all other options as "no". Frequency of *alcohol consumption* past year was assessed by five categories. *Body Mass Index* (BMI) was based on self-reported data and did not differ between distressed and non-distressed men and women, respectively. As there was no significant age-gender adjusted association between BMI and mortality in our study sample, BMI was excluded from the multiadjusted analyses. *Chronic disease* was assessed by the question: "Do you have any long-term disease, injury-related trouble, disability or other weakness?" (yes/no).

Statistics

Chi-square and *t* tests were used to compare the sample characteristic differences between non-distressed/ distressed, men and women respectively (cut-off GHQ 2/3) (Table 1).

As the main aim of the study was to test gender differences, an initial test for effect modification by gender was performed. The interaction term between psychological distress and sex was significant (Odds Ratio (OR) = 0.5; P = 0.008) indicating that the effect of psychological distress on mortality was different for men and women.

The P-value for proportionality based on interaction term between the variable psychological distress and time of follow-up was <.001 indicating non-fulfillment of the proportional hazards assumption. Logistic regression models were used to compute study specific odds

For peer review only - http://bmjopen.bmj.com/site/about/guidelines.xhtml

ratios with accompanying 95 % confidence intervals for the association between distress and all-cause mortality, as well as the association between distress and death caused by cardiovascular disease, cancer, or other causes, respectively. Model 0 adjusted for age and gender, Model 1 furthermore adjusted for SES, low physical activity, daily smoking, and alcohol consumption, and Model 2 furthermore adjusted for chronic disease. Table 2 shows logistic regression models for women and men combined. Table 3 shows logistic regression models stratified by gender (Model 0 age-adjusted only). Table 4 shows overall and gender-stratified associations between different levels of psychological distress and all-cause mortality (GHQ score: 0, 1-2, 3-5, 6-12). The statistical analyses were performed using SAS 9.4, and all analyses were performed on weighted data. To ensure appropriate variance estimation on weighted data, we used bootstrap methods with 2000 numbers of replicates to obtain confidence intervals and p-values. The survey means procedure was used to obtain weighted descriptive statistics for continuous variables, and the survey frequency procedure was used for weighted one-way and multi-way crosstabulations. The Surveylogistic was used for weighted logistic regression analysis.[21] In order to compare two different cut-off scores, identical statistical analyses using cut-off GHQ 3/4 were furthermore performed (see Supplementary Tables 1-4).

RESULTS

At baseline, more women than men were psychologically distressed, 20.2 % compared to 15.7 % (Table 1). Distressed study members were in average younger, more likely to not belong to the workforce, more likely to be sedentary, to smoke and to have a chronic disease. As can be seen by non-overlapping confidence intervals, distressed women were significantly younger, leaner, less sedentary, and drank alcohol less often than distressed men.

For peer review only - http://bmjopen.bmj.com/site/about/guidelines.xhtml

Table 1. Descriptive characteristics (%) of age, SES, sedentary leisure time, daily smoking, alcohol, BMI, and chronic distress by psychological distress (GHQ-12 ≥3).
The 2008 Public Health Survey of Scania, Sweden. Total population n=25503. Weighted prevalence.

	**Women** n = 13984 **Psychological distress**			**Men** n = 11519 **Psychological distress**		
	**No** n = 11452 79.8 %	**Yes** n = 2532 **20.2 %**	p-value	**No** n = 9925 84.3 %	**Yes** n = 1594 **15.7 %**	p-value
**Age**, yrs: mean ± SD [a]	46.9 ± 15.9 (46.5-47.3)	40.6 ± 16.6 (39.8-41.4)	<0.001	47.5 ± 17.6 (47.0-47.9)	43.4± 17.5 (42.4-44.3)	<0.001
**Body Mass Index (BMI)**: mean ± SD [a]	24.9 ± 4.6 (24.8-25.1)	24.9 ± 5.2 (24.7-25.2)	0.947	26.1 ± 4.1 (26.0-26.2)	26.3 ± 5.0 (26.0-26.6)	0.165
**Socioeconomic status (SES)** [b]						
Higher non-manual	7.8 (7.3-8.4)	7.2 (6.0-8.3)		9.9 (9.2-10.6)	9.0 (7.3-10.7)	
Medium non-manual	15.9 (15.1-16.7)	13.6 (11.9-15.2)		12.3 (11.5-13.2)	9.3 (7.8-10.9)	
Lower non-manual	10.6 (10.0-11.3)	9.6 (8.2-11.0)		5.3 (4.7-5.9)	5.3 (3.9-6.6)	
Skilled manual	9.6 (8.9-10.3)	7.7 (6.4-9.0)		11.8 (11.0-12.6)	11.3 (9.3-13.3)	
Unskilled manual	12.4 (11.6-13.2)	11.5 (10.0-12.9)		13.5 (12.6-14.4)	9.8 (8.1-11.2)	
Self-employed/farmer	4.1 (3.7-4.6)	3.2 (2.3-4.0)		8.4 (7.8-9.1)	6.1 (4.8-7.4)	
Early retired	3.9 (3.5-4.3)	6.7 (5.5-7.9)		2.6 (2.2-3.0)	8.7 (6.9-10.6)	
Unemployed	3.1 (2.6-3.6)	8.8 (7.3-10.2)		2.9 (2.4-3.3)	9.3 (7.4-11.2)	
Student	8.5 (7.8-9.2)	13.5 (11.7-15.4)		6.4 (5.7-7.1)	9.8 (7.8-11.7)	
Old age pensioner	19.1 (18.2-19.9)	9.7 (8.3-11.0)		20.3 (19.5-21.2)	10.0 (8.4-11.5)	
Unclassified	4.2 (3.6-4.7)	4.6 (3.5-5.7)		6.1 (5.4-6.8)	7.9 (6.0-9.7)	
Long-term sickleave	0.7 (0.5-0.9)	4.0 (3.0-5.0)	<0.001	0.4 (0.3-0.6)	3.5 (2.5-4.6)	<0.001
**Sedentary leisure time** [b]	11.0 (10.3-11.8)	19.8 (17.8-21.8)	<0.001	13.4 (12.6-14.3)	27.1 (24.3-29.9)	<0.001
**Daily smoking** [b]	14.6 (13.8–15.4)	19.2 (17.2-21.2)	<0.001	12.5 (11.6-13.3)	21.0 (18.3-23.7)	<0.001
**Alcohol drinking past year**[b]						
Never	14.0 (13.1-14.8)	18.6 (16.7-20.6)		8.6 (7.9-9.3)	13.6 (11.3-15.9)	
Once a month or more seldom	27.2 (26.2-28.2)	27.8 (25.6-30.0)		18.2 (17.2-19.1)	22.7 (20.0-25.3)	
2-4 times a month	34.4 (33.3-35.5)	32.8 (30.6-35.1)		37.7 (36.5-38.8)	32.3 (29.4-35.2)	
2-3 times a week	20.0 (19.1-20.9)	15.2 (13.5-16.9)		26.1 (25.0-27.1)	22.0 (19.4-24.5)	
At least 4 times a week	4.5 (4.0-4.9)	5.6 (4.6-6.6)	<0.001	9.5 (8.9-10.2)	9.4 (7.8-11.1)	<0.001
**Chronic disease*** [b]	26.5 (25.5-27.4)	43.1 (40.7-45.5)	<0.001	24.3 (23.2-25.3)	45.5 (42.4-48.5)	<0.001

GHQ-12 = Twelve item version of the General Health Questionnaire, 0-12 points. Psychological distress was defined as GHQ-12 ≥3.
* Chronic disease = long-term disease, injury-related trouble, disability or other weakness.
[a] p-value: Independent samples T-test, 2-tailed
[b] p-value: Pearson Chi Square test, 2-sided
The values in parentheses are 95% confidence intervals for mean or percent based on bootstrap method with 2000 number of replicates.

For peer review only - http://bmjopen.bmj.com/site/about/guidelines.xhtml

The mortality rates (# of deaths per 100 person-years) were calculated for men and women (data not shown). The overall mortality rate was 0.88 (95% CI: 0.82-0.95) for men and 0.50 (0.47-0.55) for women, with a male to female Incidence Rate Ratio (IRR) of 1.75 (95% CI: 1.57-1.95; p-value <0.001). The male to female IRR for cause-specific mortality were as follows: cardiovascular causes 2.51 (2.05-3.07; <0.001), cancer 1.34 (1.13-1.59; <0.001), and "other causes" 1.74 (1.44 – 2.11; <0.001), with non-overlapping 95% confidence intervals for men and women, respectively.

Table 2 shows associations between psychological distress and mortality (all-cause and cause-specific) in multi-adjusted logistic regression analyses for men and women combined. Age and gender-adjusted associations remained significant through Model 1 (SES, physical activity, smoking, alcohol) and Model 2 (chronic disease) for all mortality end-points except cancer. For all-cause mortality, the initial age-and gender adjusted OR 2.8 (95 % CI: 2.3 – 3.4) decreased to 1.8 (1.4 – 2.2) in the final model.

Table 2. ORs from logistic regression models for all-cause mortality and cause-specific mortality, showing association with psychological distress (GHQ ≥3).
The 2008 Scania public health survey with 8.3 years follow-up.
Men and women combined; n = 25503.

	Model 0		Model 1		Model 2		
**Cause of death**	**OR**	**(95%CI)**	**OR**	**(95%CI)**	**OR**	**(95%CI)**	**Number of deaths**
**All causes**	**2.8*** **	(2.3-3.4)	**2.1*** **	(1.7-2.6)	**1.8*** **	(1.4-2.2)	1389
**Cause-specific:**							
**Cardiovascular**	**2.9*** **	(2.1-4.1)	**2.2*** **	(1.5-3.1)	**1.8*** **	(1.3-2.6)	425
**Cancer**	**1.7*** **	(1.3-2.3)	**1.5* **	(1.1-2.0)	1.3	(1.0-1.8)	539
**Other causes**	**2.8*** **	(2.0-3.8)	**1.9*** **	(1.4-2.6)	**1.6** **	(1.2-2.2)	425

Model 0 adjusted for age.
Model 1 furthermore adjusted for socioeconomic status, physical activity, smoking, and alcohol.
Model 2 furthermore adjusted for chronic disease.
Significance levels: * p<0.05, ** p<0.01, *** p<0.001
Weighted Odds Ratios. Bootstrap method (2000 replicates) for variation estimation.

For peer review only - http://bmjopen.bmj.com/site/about/guidelines.xhtml

Gender stratification revealed a clear difference (Table 3). Among men the association between psychological distress and mortality remained robust through the modelling for all mortality-outcomes except cancer. Among women, the association was only significant through the modelling for all-cause mortality. The age-adjusted odds ratio for all-cause mortality decreased from 3.3 (2.5 – 4.4) to 2.1 (1.6 – 2.9) in men, and from 2.3 (1.7 – 3.0) to 1.4 (1.0 – 1.8) in women after full adjustments.

Table 3. ORs from logistic regression models for all-cause mortality and cause-specific mortality, showing association with psychological distress (GHQ ≥3).
The 2008 Scania public health survey with 8.3 years follow-up.
Stratified by gender; n = 13984 women and 11519 men.

	Model 0		Model 1		Model 2		
**Cause of death**	**OR**	**(95%CI)**	**OR**	**(95%CI)**	**OR**	**(95%CI)**	**Number of deaths**
**All causes**							
Women	**2.3*** **	(1.7-3.0)	**1.6** **	(1.2-2.1)	**1.4***	(1.0-1.8)	574
Men	**3.3*** **	(2.5-4.4)	**2.6*** **	(1.9-3.5)	**2.1*** **	(1.6-2.9)	815
**Cause-specific:**							
**Cardiovascular**							
Women	**2.5*** **	(1.5-4.2)	1.7	(1.0-2.8)	1.4	(0.8-2.4)	140
Men	**3.1*** **	(2.1-4.8)	**2.5*** **	(1.5-4.0)	**2.1** **	(1.3-3.3)	285
**Cancer**							
Women	**1.5***	(1.0-2.3)	1.2	(0.8-1.9)	1.1	(0.7-1.7)	258
Men	**2.0*** **	(1.3-3.0)	**1.7***	(1.1-2.7)	1.5	(1.0-2.3)	281
**Other causes**							
Women	**2.4*** **	(1.6-3.8)	**1.6***	(1.0-2.5)	1.3	(0.8-2.1)	176
Men	**3.0*** **	(2.0-4.5)	**2.2*** **	(1.4-3.3)	**1.8***	(1.1-2.8)	249

Model 0 adjusted for age.
Model 1 furthermore adjusted for socioeconomic status, physical activity, smoking, and alcohol.
Model 2 furthermore adjusted for chronic disease.
Significance levels: * p<0.05, ** p<0.01, *** p<0.001
Weighted Odds Ratios. Bootstrap method (2000 replicates) for variation estimation.

In order to investigate a dose-response pattern in the association between GHQ-12 and all-cause mortality, the study population was divided into four groups according to GHQ-score: 0 no distress = reference category (69 %), 1-2 (15 %), 3-5 (8 %), and 6-12 (8 %). A clear pattern of increasing mortality by increasing distress was seen for men and women combined in the fully adjusted logistic regression analyses (Table 4). Gender stratification showed that this was mainly due to a strong dose-response pattern among men.

For peer review only - http://bmjopen.bmj.com/site/about/guidelines.xhtml

Table 4. Associations between different levels of psychological distress (GHQ-12) and all-cause mortality.
The 2008 Scania public health survey with 8.3 years follow-up.
Total population (n=25503) and stratified by gender (n=13984 women and 11519 men).

GHQ-score	Model 0 OR	(95%CI)	Model 1 OR	(95%CI)	Model 2 OR	(95%CI)	Number of deaths
**Total population**							
0=REF	1.0		1.0		1.0		1389
1-2	**1.8*** **	(1.4-2.2)	**1.5*** **	(1.2-1.9)	**1.4** **	(1.1-1.7)	
3-5	**2.4*** **	(1.8-3.2)	**1.9*** **	(1.4-2.6)	**1.6** **	(1.2-2.1)	
6-12	**3.8*** **	(3.0-5.0)	**2.7*** **	(2.0-3.5)	**2.2*** **	(1.6-2.8)	
**Women**							574
0=REF	1.0		1.0		1.0		
1-2	**1.8*** **	(1.3-2.4)	**1.4* **	(1.0-2.0)	1.3	(0.9-1.8)	
3-5	**2.1*** **	(1.5-3.2)	**1.6* **	(1.1-2.4)	1.4	(0.9-2.0)	
6-12	**2.9*** **	(2.0-4.1)	**1.9*** **	(1.3-2.7)	**1.6* **	(1.1-2.2)	
**Men**							815
0=REF	1.0		1.0		1.0		
1-2	**1.8*** **	(1.3-2.4)	**1.5** **	(1.1-2.1)	**1.4* **	(1.0-1.9)	
3-5	**2.6*** **	(1.7-3.8)	**2.1*** **	(1.4-3.3)	**1.8** **	(1.2-2.7)	
6-12	**4.7*** **	(3.3-6.7)	**3.4*** **	(2.3-5.0)	**2.7*** **	(1.8-4.0)	

Model 0 adjusted for age (and gender in analysis of total population).
Model 1 furthermore adjusted for socioeconomic status, physical activity, smoking, and alcohol.
Model 2 furthermore adjusted for chronic disease.
Significance levels: * $p<0.05$, ** $p<0.01$, *** $p<0.001$
Weighted Odds Ratios. Bootstrap method (2000 replicates) for variation estimation.

DISCUSSION

The present study showed that the association between psychological distress measured by GHQ-12 and 8-year mortality was stronger and more robust in men than women. Among men, the association held through all adjustments (SES, life-style factors and chronic disease) for all-cause mortality, cardiovascular mortality and mortality from other causes with the exception of cancer. Among women, the association held through all adjustments for all-cause mortality only. These results are concordant with those of an earlier Finnish longitudinal study on GHQ-12 and 11-year all-cause mortality, i.e. the increased mortality risk was mainly explained by excess mortality among distressed men.[9] As previously shown, the association between GHQ-12 and mortality was weakest for cancer,[10, 11] and evidence for reverse causality was not found with the possible exception of cancer.[8, 10, 11, 13, 14]

For peer review only - http://bmjopen.bmj.com/site/about/guidelines.xhtml

For peer review only

The gender morbidity-survival paradox was illustrated in this study, as morbidity (psychological distress) was higher among women, while mortality was higher among men. Several potential explanations could be considered. To begin with, the definition of psychological distress could be gendered per se,[22] as expressions of mental ill-health may differ between men and women.[17] Men's and women's overall mental health could be similar, although expressed as different conditions - depression may be thought of as a female problem because more women seek help for depression, but more men commit suicide and turn to drinking and drug-abuse to alleviate symptoms of mental ill-health.[17] The GHQ-12 asks for symptoms of anxiety, depression, sleep disturbance, loss of confidence and the ability to perform daily activities and cope with everyday problems. Men may find it more difficult than women to recognize and report symptoms involving vulnerability because of masculine gender norms,[22] with an ensuing higher threshold to report psychological symptoms on questionnaires. Consequently, the men who do report symptoms might have a more severe disorder as suggested by their heightened mortality risk.[23] Gender is a ubiquitous aspect of mental health, present in contexts ranging from formal diagnostic criteria to the ways individuals label, communicate, and cope with their problems.[22] A more ruminative coping style among women may contribute to an increased vulnerability to depression, but women are also more willing to adopt a sick role, seek help and access health care, which facilitates earlier detection.[20, 24] Clinical depression is about twice as common in women as in men.[24] Sex differences in depression begin during early adolescence and are most pronounced during the reproductive years, and a possible biological explanation to female susceptibility to depression could be associated with estrogen and progesterone.[17, 24] The heritability of depression is also greater in women than men. Sex differences in monoamine functioning have been demonstrated (e.g. a decrease in serotonin transmission increased

For peer review only - http://bmjopen.bmj.com/site/about/guidelines.xhtml

depression significantly more in women than men), and women also respond better to antidepressive pharmacological treatment with SSRIs (Selective serotonin reuptake inhibitors) than men.[24] Furthermore, economic explanations and environmental factors may contribute to the gender difference in depression prevalence, such as a female disadvantage of lower socioeconomic status, gender pay gap, strain from dual (work-family) roles and higher rates of exposure to IPV (intimate partner violence).[18, 24]

A recent study investigated psychological distress and heart disease mortality in the United States, with data from more than half a million participants in the National Health Interview Survey (NHIS) and 18 years of mortality follow-up.[25] Results showed a robust dose-response relationship between psychological distress (measured by Kessler 6 item psychological distress scale, K-6) and heart disease mortality, indicating psychological distress to be an important predictor of heart disease mortality. The relative risk of heart disease mortality associated with psychological distress was higher among men than women, in line with results from the present study.[25] Another study highlighted the importance of identifying men with psychological distress when assessing CVD risk, as psychological distress was associated with higher cardiovascular risk scores among men but not women.[26] Psychological distress may increase the risk of heart disease through various mechanisms, e.g. promoting inflammatory processes, and activating automatic nerve system or hypothalamic-pituitary-adrenal (HPA) influences.[25] Psychological distress is also highly correlated with traditional CVD risk factors such as smoking, physical inactivity, obesity, hypertension, and adherence to medical treatment.[12, 25] Further research is needed to address pathways through which psychological distress increases mortality and how different underpinning pathways will be responsible for gendered patterns of morbidity and mortality in different health outcomes.[18, 19, 25] The underlying mechanisms in gender differences need to be better understood in order to develop effective public-health and clinical interventions. Our

For peer review only - http://bmjopen.bmj.com/site/about/guidelines.xhtml

study supports the validity of GHQ-12 as a predictor of mortality primarily among men. It has been suggested that GHQ-12 might possibly be an even stronger predictor of mortality than CIDI-diagnosed mood/anxiety disorders, since diagnosed mental disorders tend to be treated whereas self-reported psychological distress does not.[11] In the future, it would be valuable to validate screening tools for psychological distress separately for men and women.[24]

A minor additional aim of the present study was to compare two different GHQ-12 cut-off scores: GHQ-12 ≥3 (conventionally used in Sweden) and GHQ-12 ≥4 (widely used internationally). The choice of a cut-off point depends on the purpose of the study, and a recent Swedish case-control study concluded that although the cut-off points 2-4 were all deemed acceptable, the best sensitivity and specificity of GHQ-12 when discriminating between healthy controls and psychiatric outpatients was seen at cut-off ≥4.[7] In the present study GHQ-12 ≥3 defined 17.9 % of the study population as psychologically distressed (women: 20.2 %; men: 15.7 %), while GHQ-12 ≥4 defined 14.2 % as psychologically distressed (women: 16.2 %; men: 12.1 %) (Supplementary Table 1). The higher cut-off led to generally slightly stronger associations with mortality (Supplementary Table 2-3). A higher cut-off led to a slightly more pronounced dose-response pattern for all-cause mortality in men but a less consistent pattern in women (Supplementary Table 4).

Strengths and limitations

This was a large prospective community study based on a stratified randomized sample of people in Scania 18-80 years old who participated in the 2008 Scania Public Health Survey. The response rate of 54 % is a limitation, but a weighting variable was specifically designed to compensate for self selection bias in the statistical analyses. Non-responders were more often young, male, low-educated or foreign-born. We do not know if non-responders had

For peer review only - http://bmjopen.bmj.com/site/about/guidelines.xhtml

more psychological distress than responders, but there were probably more non-responders among persons with serious psychiatric disease (such as psychosis, schizophrenia, severe depression) who are known to have a higher risk of premature death [27] than in the general population, due to the mental task involved in answering a large survey with 134 main questions (totalling 273 items including subqueries and follow-up questions). Most base-line data was self-reported and our study lacked objective measures of health such as blood pressure, verified physical/ mental illness and biomarkers. Among those who died in our study population, more than 50 % reported baseline chronic disease (data not shown). We had no objective measure of pre-existing disease at baseline, and self-reported chronic disease may not be a valid indicator of pre-existing disease. The mortality rates across age strata were similar to national data.[28] Psychological distress was measured at baseline only, and the persistence of distress may be an important determinant of mortality risk.[29] With an instrument such as the GHQ-12 asking for symptoms during "the past few weeks" a smaller portion of persons with short-term/ intermittent than long-term/ persistent psychological distress will be identified at baseline. Over time, some of the participants defined as cases at baseline will recover while some defined as non-cases will develop psychological distress, a form of misclassification diluting the effect of psychological distress on mortality over time (length-time bias).[13, 30, 31] This phenomenon was clearly illustrated by a large prospective study investigating the temporal robustness of psychological distress (Kessler 6 items, K-6) by re-estimating the mortality impact at 2-year, 5-year, and 10-year follow-up times.[25] Data used in the present study did not fulfil the proportional assumption. Logistic regression analysis was therefore used instead of cox regression survival analysis, the method of choice in previous studies.[2, 8-16, 23, 25, 29] However, additional analyses showed that the results from Cox regression analysis and logistic regression analysis were fairly similar in magnitude (data not shown).

For peer review only - http://bmjopen.bmj.com/site/about/guidelines.xhtml

Conclusions

The present study extends previous research by investigating gender differences in the association between psychological distress measured by GHQ-12 and mortality. Results showed strong and robust associations in men but not in women. GHQ-12 could be used as one of several predictors of mortality, especially among men. The results of the present study are probably mainly generalizable to other western countries, as both exposure and outcome are highly internationally validated measures, but the mediating factors between psychological distress and mortality may be more culturally diverse.

Contributorship statement: ML and MF designed the study in collaboration with MR. ML assembled the database. MP analysed the data with input from ML and MF. MF wrote the first draft. All authors contributed to the paper and approved the final draft.

Competing interests: None declared.

Funding: This work was supported by the Swedish Research Council (Vetenskapsrådet) [K2014-69X-22427-01-4] and the Swedish ALF Government [Grant Dnr F 2014/354].

Data sharing statement: No additional data available.

Acknowledgements: The authors wish to thank all participants for their cooperation, and Region Skåne (Scania) for providing data from the 2008 Scania public health survey.

For peer review only

REFERENCES

1. Calling S, Midlöv P, Johansson S-E, *et al*. Longitudinal trends in self-reported anxiety. Effects of age and birth cohort during 25 years. *BMC Psychiatry* 2017;17:119. doi: 10.1186/s12888-017-1277-3.

2. Chiu M, Vigod S, Rahman F, *et al*. Mortality risk associated with psychological distress and major depression: A population-based cohort study. *J Affect Disord* 2018;234:117-23. doi: 10.1016/j.jad.2018.02.075.

3. Pedersen SS, von Känel R, Tully PJ, *et al.* Psychosocial perspectives in cardiovascular disease. *Eur J Prev Cardiol* 2017;24(3):108-115. doi: 10.1177/2047487317703827.

4. Bortolato B, Hyphantis TN, Valpione S, *et al.* Depression in cancer: The many biobehavioral pathways driving tumor progression. *Cancer Treat Rev* 2017;52:58-70. doi: 10.1016/j.ctrv.2016.11.004.

5. Goldberg DP, Gater R, Sartorius N, *et al*. The validity of two versions of the GHQ in the WHO study of mental illness in general health care. *Psychol Med* 1997;27:191-7. doi: 10.1017/s0033291796004242.

6. McDowell I. Measuring health: A guide to rating scales and questionnaires. 3rd edition. New York: Oxford University Press 2006:259-72.

For peer review only

For peer review only - http://bmjopen.bmj.com/site/about/guidelines.xhtml

7. Lundin A, Åhs J, Åsbring N, *et al*. Discriminant validity of the 12-item version of the general health questionnaire in a Swedish case-control study. *Nord J Psychiatry* 2017;71:171-9. doi: 10.1080/08039488.2016.1246608.

8. Robinson KL, McBeth J, Macfarlane GJ. Psychological distress and premature mortality in the general population: A prospective study. *Ann Epidemiol* 2004;14:467-72. doi: 10.1016/j.annepidem.2003.11.007.

9. Puustinen PJ, Koponen H, Kautiainen H, *et al*. Psychological distress measured by the GHQ-12 and mortality: A prospective population-based study. *Scand J Public Health* 2011;39(6):577-81. doi: 10.1177/1403494811414244.

10. Russ TC, Stamatakis E, Hamer M, *et al*. Association between psychological distress and mortality: individual participants pooled analysis of 10 prospective cohort studies. *BMJ* 2012;345:e4933. doi: 10.1136/bmj.e4933.

11. Ponizovsky AM, Haklai Z, Goldberger N. Association between psychological distress and mortality: the case of Israel. *J Epidemiol Community Health* 2018;72(8):726-32. doi: 10.1136/jech-2017-210356.

For peer review only

12. Hamer M, Molloy GJ, Stamatakis E. Psychological distress as a risk factor for cardiovascular events: pathophysiological and behavioral mechanisms. *J Am Coll Cardiol* 2008;52(25):2156-62. doi: 10.1016/j.jacc.2008.08.057.

13. Hamer M, Kivimaki M, Stamatakis E, *et a*l. Psychological distress as a risk factor for death from cerebrovascular disease. *CMAJ* 2012;184(13):1461-6. doi: 10.1503/cmaj.111719.

14. Batty GD, Russ TC, Stamatakis E, *et al*. Psychological distress and risk of peripheral vascular disease, abdominal aortic aneurysm, and heart failure: Pooling of sixteen cohort studies. *Atherosclerosis* 2014;236(2):385-8. doi: 10.1016/j.atherosclerosis.2014.06.025.

15. Hamer M, Chida Y, Molloy GJ. Psychological distress and cancer mortality. *J Psychosom Res* 2009;66(3):255-8. doi: 10.1016/j.jpsychores.2008.11.002.

16. Batty GD, Russ TC, Stamatakis E, *et al*. Psychological distress in relation to site specific cancer mortality: pooling of unpublished data from 16 prospective cohort studies. *BMJ* 2017;356:j108. doi: 10.1136/bmj.j108.

17. Rieker PP, Bird CE. Rethinking gender differences in health: Why we need to integrate social and biological perspectives. *J Gerontol B Psychol Sci Soc Sci* 2005;60(2):4-7. doi: 10.1093/geronb/60.special_issue_2.s40.

For peer review only

For peer review only - http://bmjopen.bmj.com/site/about/guidelines.xhtml

18. Bambra C, Albani V, Franklin P. COVID-19 and the gender health paradox. *Scand J Public Health* 2021;49(1):17-26. doi: 10.1177/1403494820975604.

19. Oksuzyan A, Brønnum-Hansen H, Jeune B. Gender gap in health expectancy. *Eur J Ageing* 2010;7(4):213-8. doi: 10.1007/s10433-010-0170-4.

20. Gordon EH, Hubbard RE. Do sex differences in chronic disease underpin the sex-frality paradox? *Mech Ageing Dev* 2019;179:44-50. doi: 10.1016/j.mad.2019.02.004.

21. SAS/STAT Software Survey Analysis https://support.sas.com/rnd/app/stat/procedures/SurveyAnalysis.html (accessed 31 July 2021).

22. Addis ME, Hoffman E. Men's depression and help-seeking through the lenses of gender. In: Levant RF, Wong YJ, eds. The Psychology of Men and Masculinities. American Psychological Association 2017:171-196.

23. Gunnell D, Rasul F, Stansfeld SA, *et al*. Gender differences in self-reported minor mental disorder and its association with suicide. A 20-year follow-up of the Renfrew and Paisley cohort. *Soc Psychiatry Psychiatr Epidemiol* 2002;37(10):457–9. doi: 10.1007/s00127-002-0579-y.

24. Shah BM, Kornstein SG. Mental health: Sex and gender evidence in depression, general anxiety disorder, and schizophrenia. In: Jenkins MR, Newman CB, eds. How sex and gender impact clinical practice. An evidence-based guide to patient care. Elsevier and Academic Press 2021:153-169.

For peer review only - http://bmjopen.bmj.com/site/about/guidelines.xhtml

For peer review only

25. Lee H, Singh GK. Psychological distress and heart disease mortality in the United States: Results from the 1997-2014 NHIS-NDI Record Linkage Study. *Int J MCH AIDS* 2020;9(3):260-73. doi: 10.21106/ijma.391.

26. Puustinen PJ, Koponen H, Kautiainen H, *et al*. Gender-specific association of psychological distress with cardiovascular risk scores. *Scand J Prim Health Care* 2010; 28(1):36-40. doi: 10.3109/02813431003648131.

27. Ösby U, Westman J, Hällgren J, *et al*. Mortality trends in cardiovascular causes in schizophrenia, bipolar and unipolar mood disorder in Sweden 1987-2010. *Eur J Public Health* 2016;26(5):867-71. doi: 10.1093/eurpub/ckv245.

28. The Public Health Agency (Folkhälsomyndigheten). Public health in Sweden 2016. Annual report. (Folkhälsan i Sverige 2016. Årlig rapportering.) In Swedish. Available from: https://www.folkhalsomyndigheten.se/contentassets/cc89748e004743c39ff4c03fec24c570/folkhalsan-i-sverige-2016-16005.pdf (accessed 14 July 2021).

29. Stewart RAH, Colquhoun DM, Marschner SL, *et al*. Persistent psychological distress and mortality in patients with stable coronary artery disease. *Heart* 2017;103(23):1860-6. doi: 10.1136/heartjnl-2016-311097.

For peer review only

For peer review only - http://bmjopen.bmj.com/site/about/guidelines.xhtml

30. Rothman, K. Epidemiology. An Introduction. Oxford, New York: Oxford University Press 2002:204.

31. Aschengrau A, Seage III GR. Epidemiology in Public Health. Third Edition. Burlington, MA: Jones and Bartlett Learning 2014:442.

r peer review only

For peer review only - http://bmjopen.bmj.com/site/about/guidelines.xhtml

Supplementary Table 1. Descriptive characteristics (%) of age, SES, sedentary leisure time, daily smoking, alcohol, BMI, and chronic distress by psychological distress (GHQ-12 ≥4).
The 2008 Public Health Survey of Scania, Sweden. Total population n=25503. Weighted prevalence.

	**Women** n = 13984 Psychological distress			**Men** n = 11519 Psychological distress		
	**No** n = 11950 83.8 %	**Yes** n = 2034 **16.2 %**	p-value	**No** n = 10283 87.9 %	**Yes** n = 1236 **12.1 %**	p-value
**Age**, yrs: mean ± SD [a]	46.6 ± 16.0 (46.2-47.0)	40.6 ± 16.6 (39.7-41.4)	<0.001	47.2 ± 17.7 (46.8-47.6)	44.0± 17.5 (42.9-45.0)	<0.001
**Body Mass Index (BMI)**: mean ± SD [a]	24.9 ± 4.6 (24.8-25.1)	24.9 ± 5.3 (24.7-25.2)	0.931	26.1 ± 4.1 (26.0-26.2)	26.4 ± 5.1 (26.0-26.7)	0.102
**Socioeconomic status (SES)** [b]						
Higher non-manual	7.9 (7.3-8.4)	6.8 (5.5-8.1)		9.8 (9.1-10.5)	9.4 (7.5-11.3)	
Medium non-manual	15.7 (15.0-16.5)	13.8 (12.0-15.6)		12.3 (11.5-13.1)	8.5 (6.7-10.3)	
Lower non-manual	10.6 (9.9-11.3)	9.6 (8.1-11.2)		5.3 (4.8-5.9)	5.1 (3.6-6.6)	
Skilled manual	9.5 (8.6-10.1)	7.6 (6.1-9.0)		12.0 (11.2-12.8)	10.1 (7.9-12.3)	
Unskilled manual	12.6 (11.8-13.3)	10.5 (8.9-12.1)		13.4 (12.5-14.2)	9.6 (7.5-11.6)	
Self-employed/farmer	4.1 (3.7-4.6)	3.1 (2.1-4.1)		8.3 (7.6-9.0)	6.4 (4.8-8.0)	
Early retired	3.9 (3.5-4.4)	7.3 (5.8-8.7)		2.6 (2.2-3.0)	10.1 (7.7-12.4)	
Unemployed	3.3 (2.8-3.7)	9.3 (7.6-11.0)		3.0 (2.5-3.5)	10.1 (7.9-12.4)	
Student	8.8 (8.1-9.5)	13.2 (11.1-15.3)		6.5 (5.9-7.2)	9.9 (7.5-12.3)	
Old age pensioner	18.6 (17.8-19.4)	9.9 (8.4-11.3)		19.9 (19.0-20.8)	10.1 (8.4-11.9)	
Unclassified	4.2 (3.7-4.7)	4.5 (3.3-5.7)		6.4 (5.7-7.1)	6.6 (4.6-8.5)	
Long-term sickleave	0.8 (0.6-1.0)	4.4 (3.3-5.6)	<0.001	0.5 (0.3-0.6)	4.2 (2.9-5.5)	<0.001
**Sedentary leisure time** [b]	11.2 (10.4-11.9)	21.4 (19.1-23.7)	<0.001	13.7 (12.8-14.6)	28.9 (25.6-32.1)	<0.001
**Daily smoking** [b]	14.7 (13.9–15.5)	19.6 (17.5-21.8)	<0.001	12.7 (11.8-13.5)	22.1 (19.1-25.1)	<0.001
**Alcohol drinking past year**[b]						
Never	14.2 (13.3-15.0)	18.6 (16.5-20.8)		8.6 (7.9-9.4)	14.8 (12.1-17.5)	
Once a month or more seldom	27.1 (26.1-28.1)	28.6 (26.1-31.0)		18.2 (17.3-19.2)	23.4 (20.4-26.5)	
2-4 times a month	34.6 (33.5-35.6)	31.5 (28.9-34.0)		37.8 (36.7-39.0)	29.5 (26.3-32.6)	
2-3 times a week	19.7 (18.9-20.6)	15.3 (13.5-17.2)		25.8 (24.8-26.8)	22.5 (19.6-25.4)	
At least 4 times a week	4.4 (4.0-4.9)	6.0 (4.8-7.1)	<0.001	9.4 (8.8-10.1)	9.9 (8.0-11.7)	<0.001
**Chronic disease*** [b]	26.8 (25.9-27.8)	45.3 (42.7-48.0)	<0.001	24.8 (23.8-25.8)	47.8 (44.4-51.2)	<0.001

GHQ-12 = 12 item version of the General Health Questionnaire, 0-12 points. Psychological distress was defined as GHQ-12≥4.
* Chronic disease = long-term disease, injury-related trouble, disability or other weakness.
[a] p-value: Independent samples T-test, 2-tailed
[b] p-value: Pearson Chi Square test, 2-sided
The values in parentheses are 95% confidence intervals for mean or percent based on bootstrap method with 2000 number of replicates.

For peer review only

Suppl Table 2. ORs from logistic regression models for all-cause mortality and cause-specific mortality, showing association with psychological distress (GHQ ≥4).
The 2008 Scania public health survey with 8.3 years follow-up.
Men and women combined; n = 25503.

	Model 0		Model 1		Model 2		
**Cause of death**	**OR**	**(95%CI)**	**OR**	**(95%CI)**	**OR**	**(95%CI)**	**Number of deaths**
**All causes**	**3.0*** **	(2.4-3.8)	**2.2*** **	(1.8-2.8)	**1.8*** **	(1.5-2.3)	1389
**Cause-specific:**							
**Cardiovascular**	**3.1*** **	(2.2-4.4)	**2.2*** **	(1.5-3.3)	**1.9*** **	(1.3-2.8)	425
**Cancer**	**1.8*** **	(1.3-2.4)	**1.5***	(1.1-2.1)	1.3	(0.9-1.8)	539
**Other causes**	**3.1*** **	(2.2-4.2)	**2.1*** **	(1.5-2.9)	**1.7****	(1.2-2.4)	425

Model 0 adjusted for age and gender.
Model 1 furthermore adjusted for socioeconomic status, physical activity, smoking, and alcohol.
Model 2 furthermore adjusted for chronic disease.
Significance levels: * p<0.05, ** p<0.01, *** p<0.001
Weighted Odds Ratios. Bootstrap method (2000 replicates) for variation estimation.

For peer review only

Suppl Table 3. ORs from logistic regression models for all-cause mortality and cause-specific mortality, showing association with psychological distress (GHQ ≥4).
The 2008 Scania public health survey with 8.3 years follow-up.
Stratified by gender; n = 13984 women and 11519 men.

	Model 0		Model 1		Model 2		
**Cause of death**	**OR**	**(95%CI)**	**OR**	**(95%CI)**	**OR**	**(95%CI)**	**Number of deaths**
**All causes**							
Women	**2.5*** **	(1.9-3.4)	**1.7** **	(1.2-2.3)	**1.4***	(1.1-2.0)	574
Men	**3.5*** **	(2.6-4.7)	**2.7*** **	(2.0-3.7)	**2.2*** **	(1.6-3.0)	815
**Cause-specific:**							
**Cardiovascular**							
Women	**2.7*** **	(1.6-4.6)	1.7	(1.0-2.9)	1.4	(0.8-2.5)	140
Men	**3.3*** **	(2.1-5.2)	**2.5*** **	(1.5-4.2)	**2.1** **	(1.3-3.5)	285
**Cancer**							
Women	1.5	(0.9-2.3)	1.1	(0.7-1.9)	1.0	(0.6-1.7)	258
Men	**2.1*** **	(1.3-3.3)	**1.9** **	(1.2-2.9)	**1.6***	(1.0-2.6)	281
**Other causes**							
Women	**3.0*** **	(1.9-4.8)	**1.9***	(1.2-3-0)	1.6	(1.0-2.5)	176
Men	**3.0*** **	(1.9-4.7)	**2.2*** **	(1.4-3.4)	**1.8***	(1.1-3.0)	249

Model 0 adjusted for age.
Model 1 furthermore adjusted for socioeconomic status, physical activity, smoking, and alcohol.
Model 2 furthermore adjusted for chronic disease.
Significance levels: * p<0.05, ** p<0.01, *** p<0.001
Weighted Odds Ratios. Bootstrap method (2000 replicates) for variation estimation.

For peer review only

Suppl Table 4. Associations between different levels of psychological distress (GHQ-12) and all-cause mortality.
The 2008 Scania public health survey with 8.3 years follow-up.
Total population (n=25503) and stratified by gender (n=13984 women and 11519 men).

GHQ-score	Model 0 OR	(95%CI)	Model 1 OR	(95%CI)	Model 2 OR	(95%CI)	Number of deaths
**Total population**							
0=REF	1.0		1.0		1.0		1389
1-3	**1.8*** **	(1.5-2.2)	**1.5*** **	(1.2-1.9)	**1.4** **	(1.1-1.7)	
4-6	**2.8*** **	(2.1-3.7)	**2.1*** **	(1.5-2.8)	**1.7** **	(1.3-2.4)	
7-12	**4.0*** **	(3.0-5.3)	**2.8*** **	(2.1-3.7)	**2.2*** **	(1.6-3.0)	
**Women**							
0=REF	1.0		1.0		1.0		574
1-3	**1.7*** **	(1.3-2.3)	**1.4* **	(1.0-1.9)	1.3	(0.9-1.7)	
4-6	**2.9*** **	(1.9-4.3)	**2.0** **	(1.3-3.1)	**1.7* **	(1.1-2.6)	
7-12	**2.7*** **	(1.9-4.0)	**1.7** **	(1.2-2.5)	1.4	(1.0-2.1)	
**Men**							
0=REF	1.0		1.0		1.0		815
1-3	**1.9*** **	(1.4-2.5)	**1.6*** **	(1.2-2.2)	**1.4* **	(1.1-1.9)	
4-6	**2.7*** **	(1.8-4.0)	**2.1*** **	(1.4-3.3)	**1.8** **	(1.2-2.7)	
7-12	**5.1*** **	(3.5-7.6)	**3.8*** **	(2.5-5.7)	**3.0*** **	(1.9-4.6)	

Model 0 adjusted for age (and gender in analysis of total population).
Model 1 furthermore adjusted for socioeconomic status, physical activity, smoking, and alcohol.
Model 2 furthermore adjusted for chronic disease.
Significance levels: * $p<0.05$, ** $p<0.01$, *** $p<0.001$
Weighted Odds Ratios. Bootstrap method (2000 replicates) for variation estimation.

For peer review only

STROBE Statement—Checklist of items that should be included in reports of ***cohort studies***

	Item No	Recommendation	Page No
**Title and abstract**	1	(*a*) Indicate the study's design with a commonly used term in the title or the abstract	Title
		(*b*) Provide in the abstract an informative and balanced summary of what was done and what was found	Abstract
**Introduction**			
Background/rationale	2	Explain the scientific background and rationale for the investigation being reported	3-6
Objectives	3	State specific objectives, including any prespecified hypotheses	6
**Methods**			
Study design	4	Present key elements of study design early in the paper	6-8
Setting	5	Describe the setting, locations, and relevant dates, including periods of recruitment, exposure, follow-up, and data collection	6-7
Participants	6	(*a*) Give the eligibility criteria, and the sources and methods of selection of participants. Describe methods of follow-up	6-8
		(*b*) For matched studies, give matching criteria and number of exposed and unexposed	-
Variables	7	Clearly define all outcomes, exposures, predictors, potential confounders, and effect modifiers. Give diagnostic criteria, if applicable	7-9
Data sources/ measurement	8*	For each variable of interest, give sources of data and details of methods of assessment (measurement). Describe comparability of assessment methods if there is more than one group	7-9
Bias	9	Describe any efforts to address potential sources of bias	7
Study size	10	Explain how the study size was arrived at	7
Quantitative variables	11	Explain how quantitative variables were handled in the analyses. If applicable, describe which groupings were chosen and why	7-10
Statistical methods	12	(*a*) Describe all statistical methods, including those used to control for confounding	9-10
		(*b*) Describe any methods used to examine subgroups and interactions	9-10
		(*c*) Explain how missing data were addressed	7
		(*d*) If applicable, explain how loss to follow-up was addressed	7
		(*e*) Describe any sensitivity analyses	
**Results**			
Participants	13*	(a) Report numbers of individuals at each stage of study—eg numbers potentially eligible, examined for eligibility, confirmed eligible, included in the study, completing follow-up, and analysed	7
		(b) Give reasons for non-participation at each stage	7
		(c) Consider use of a flow diagram	
Descriptive data	14*	(a) Give characteristics of study participants (eg demographic, clinical, social) and information on exposures and potential confounders	Table 1 page 11
		(b) Indicate number of participants with missing data for each variable of interest	7
		(c) Summarise follow-up time (eg, average and total amount)	8
Outcome data	15*	Report numbers of outcome events or summary measures over time	Abstract Tables 2-4

For peer review only - http://bmjopen.bmj.com/site/about/guidelines.xhtml

Main results	16	(*a*) Give unadjusted estimates and, if applicable, confounder-adjusted estimates and their precision (eg, 95% confidence interval). Make clear which confounders were adjusted for and why they were included<br>(*b*) Report category boundaries when continuous variables were categorized<br>(*c*) If relevant, consider translating estimates of relative risk into absolute risk for a meaningful time period	Table 2-4 p13-14
Other analyses	17	Report other analyses done—eg analyses of subgroups and interactions, and sensitivity analyses	9
**Discussion**			
Key results	18	Summarise key results with reference to study objectives	15
Limitations	19	Discuss limitations of the study, taking into account sources of potential bias or imprecision. Discuss both direction and magnitude of any potential bias	18-19
Interpretation	20	Give a cautious overall interpretation of results considering objectives, limitations, multiplicity of analyses, results from similar studies, and other relevant evidence	19
Generalisability	21	Discuss the generalisability (external validity) of the study results	19
**Other information**			
Funding	22	Give the source of funding and the role of the funders for the present study and, if applicable, for the original study on which the present article is based	20

*Give information separately for exposed and unexposed groups.

Note: An Explanation and Elaboration article discusses each checklist item and gives methodological background and published examples of transparent reporting. The STROBE checklist is best used in conjunction with this article (freely available on the Web sites of PLoS Medicine at http://www.plosmedicine.org/, Annals of Internal Medicine at http://www.annals.org/, and Epidemiology at http://www.epidem.com/). Information on the STROBE Initiative is available at http://www.strobe-statement.org.

For peer review only
